# Reservoir computing model of prefrontal cortex creates novel combinations of previous navigation sequences from hippocampal place-cell replay with spatial reward propagation

**DOI:** 10.1371/journal.pcbi.1006624

**Published:** 2019-07-15

**Authors:** Nicolas Cazin, Martin Llofriu Alonso, Pablo Scleidorovich Chiodi, Tatiana Pelc, Bruce Harland, Alfredo Weitzenfeld, Jean-Marc Fellous, Peter Ford Dominey

**Affiliations:** 1 INSERM, U1093, Cognition Action Plasticité Sensorimotrice, Université de Bourgogne, Dijon, France; 2 Robot Cognition Laboratory, Institut Marey, INSERM, CNRS, UBFC, Dijon, France; 3 Department of Computer Science and Engineering, University of South Florida, Tampa, Florida, United States of America; 4 Department of Psychology, University of Arizona, Tucson, Arizona, United States of America; Emory University, UNITED STATES

## Abstract

As rats learn to search for multiple sources of food or water in a complex environment, they generate increasingly efficient trajectories between reward sites. Such spatial navigation capacity involves the replay of hippocampal place-cells during awake states, generating small sequences of spatially related place-cell activity that we call “snippets”. These snippets occur primarily during sharp-wave-ripples (SWRs). Here we focus on the role of such replay events, as the animal is learning a traveling salesperson task (TSP) across multiple trials. We hypothesize that snippet replay generates synthetic data that can substantially expand and restructure the experience available and make learning more optimal. We developed a model of snippet generation that is modulated by reward, propagated in the forward and reverse directions. This implements a form of spatial credit assignment for reinforcement learning. We use a biologically motivated computational framework known as ‘reservoir computing’ to model prefrontal cortex (PFC) in sequence learning, in which large pools of prewired neural elements process information dynamically through reverberations. This PFC model consolidates snippets into larger spatial sequences that may be later recalled by subsets of the original sequences. Our simulation experiments provide neurophysiological explanations for two pertinent observations related to navigation. Reward modulation allows the system to reject non-optimal segments of experienced trajectories, and reverse replay allows the system to “learn” trajectories that it has not physically experienced, both of which significantly contribute to the TSP behavior.

## Author summary

As rats search for multiple sources of food in a complex environment, they generate increasingly efficient trajectories between reward sites, across multiple trials. This spatial navigation optimization behavior can be measured in the laboratory using a traveling salesperson task (TSP). This likely involves the coordinated replay of place-cell “snippets” between successive trials. We hypothesize that “snippets” can be used by the prefrontal cortex (PFC) to implement a form of reward-modulated reinforcement learning. Our simulation experiments provide neurophysiological explanations for two pertinent observations related to navigation. Reward modulation allows the system to reject non-optimal segments of experienced trajectories, and reverse replay allows the system to “learn” trajectories that it has not physically experienced, both of which significantly contribute to the TSP behavior.

## Introduction

Spatial navigation in the rat involves the replay of place-cell sub-sequences, that we refer to as snippets, during awake and sleep states in the hippocampus during sharp-wave-ripples (SWR) [1–4]. In the awake state, replay has been observed to take place in forward and reverse direction [2, 5–8], with respect to the physical order of the initial displacement of the animal. Both forward and reverse replay are influenced by task contingencies and reward [6, 9, 10]. Reverse replay is observed to originate from rewarded locations [11], with a greater frequency of replay for locations with greater reward, which could allow a propagation of value backwards from the rewarded location [9]. An interesting example of the impact of reward on forward replay is seen in the experiments of Gupta & van der Meer [6] where rats ran the left or right (or both) sides of a dual maze. Replay occurred with equal proportions for the same and opposite side of the rat’s current location. Same side forward replay tended to be prospective, and project forward from the current location, as observed by Pfeiffer and Foster [12]. Interestingly, opposite-side forward replay preferentially occurred retrospectively, as forward sweeps to reward locations starting from remote locations. This suggests that more diverse forward replay including forward sweeps from remote locations (as observed by Gupta & van der Meer [6]) will be observed as a function of specific task characteristics and requirements. Liu and Sibille [8] have recently shown the predictive nature of such forward sweeps, using essentially statistical data analysis techniques. Our work extends this recent work in proposing an actual mechanism and neuronal model that could support it.

Thus, while it has been observed that in both 1D [5] and 2D [12], the bulk of awake replay events are prospective, and depict future paths to upcoming goals, in more complex tasks, forward retrospective sweeps from remote locations can be observed. In the current research with multiple goal locations to be remembered (no foraging), and optimization of the paths (rather than just their memorization), we argue (and the model predicts), that forward sweeps from remote locations will predominantly carry information and be used to accomplish the task.

We focus on the role of replay during the awake state, as the animal generates increasingly efficient trajectories between reward sites, across multiple trials. This trend toward near-optimal solutions is reminiscent of the classic Traveling Salesperson Problem (TSP) [13]. The TSP problem involves finding the shortest path that visits a set of “cities” on a 2D map. It is a computationally complex problem, and is one of the most intensively studied problems in optimization [14, 15]. While it is clear that rats do not solve the TSP in the mathematical sense, they remarkably display a robust tendency towards such optimization [13]. It appears likely that such spatial navigation optimization involves planning and hence awake replay but the underlying neurophysiological mechanisms remain to be understood.

One obvious advantage of replay would be to provide additional internally generated training examples to otherwise slow reinforcement learning (RL) systems. Traditional RL methods are usually inefficient, as they use each data sample once, to incrementally improve the solution, and then discard the sample. In a real-time learning situation, where the rat is optimizing in less than ten trials, this approach is unlikely to succeed. Our model proposes to add replay to reinforcement learning to overcome this problem and improve efficiency [16]. This approach has been previously exploited with good results [17]. We will go beyond this by prioritizing replay based on a spatial gradient of reward proximity that is built up during replay. Our first hypothesis is that reward-modulated replay in hippocampus implements a simple and efficient form of reinforcement learning [18], which allows recurrent dynamics in prefrontal cortex (PFC) to consolidate snippet representations into novel efficient sequences, by rejecting sequences that are less robustly coded in the input.

**Fig 1:**
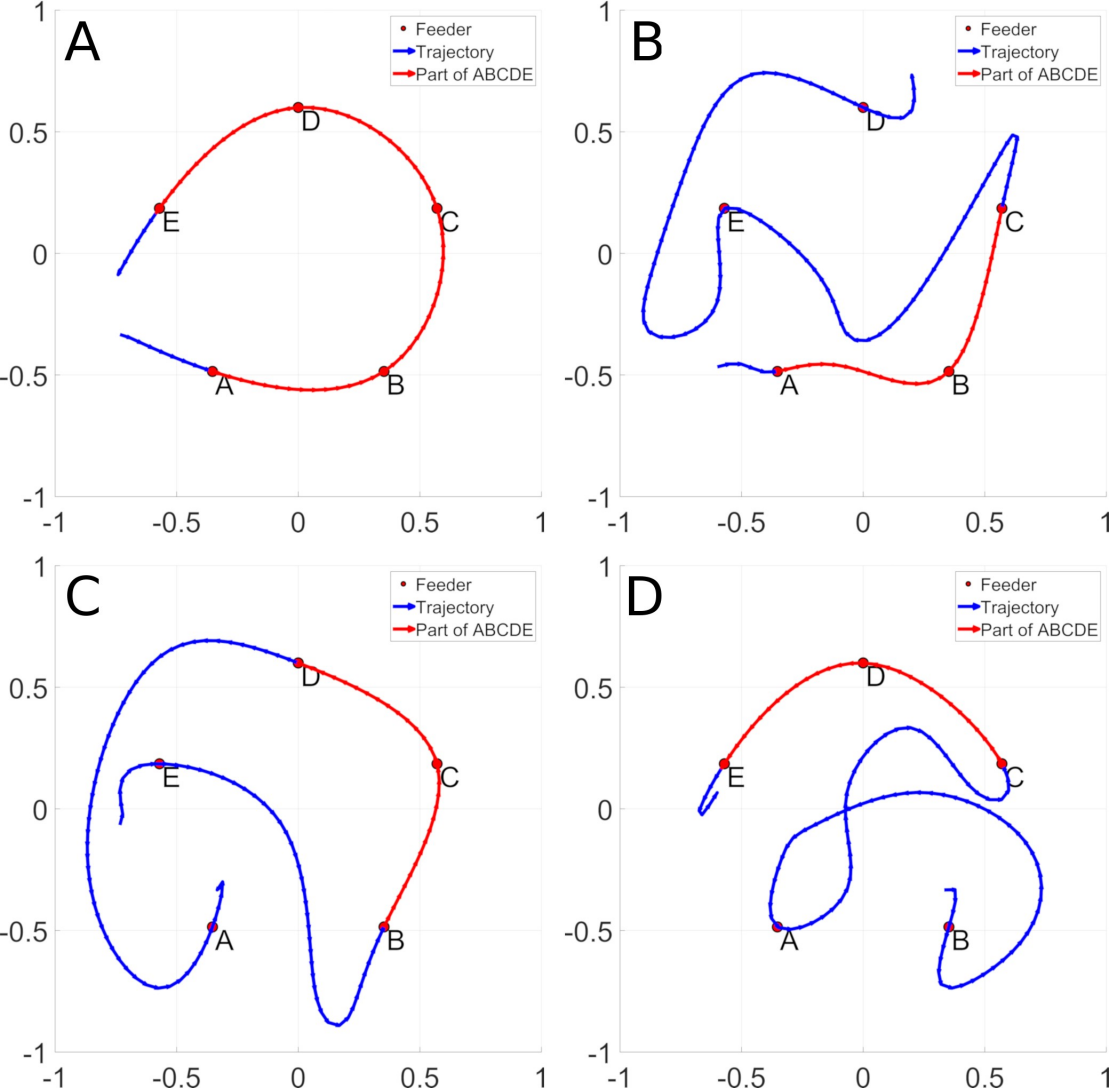
An optimal trajectory between feeders ABCDE is represented in panel A. Panel B, C and D display non optimal trajectories that contain a sub trajectory of the ABCDE trajectory. The sub trajectory shared with the ABCDE trajectory is displayed in red and the non-optimal parts in blue. Panel B contains the ABCED, panel C the EBCDA trajectory and panel D the BACDE trajectory.

An example of the behavior in question is illustrated in Fig 1. Panel A illustrates the optimal path linking the five feeders (ABCDE) in red. Panels B-D illustrate navigation trajectories that contain sub-sequences of the optimal path (in red), as well as non-optimal sub-sequences (in blue). In the framework of reward modulated replay, snippets from the efficient sub-sequences in panels B-D will be replayed more frequently, and will lead the system to autonomously generate the optimal sequence as illustrated in panel A. We thus require a sequence learning system that can re-assemble the target sequences from these replayed snippets. For this, we chose a biologically inspired recurrent network model of prefrontal cortex [19, 20] that we predict will be able to integrate snippets from examples of non-optimal trajectories and to synthesize an optimal path.

Recurrent networks have excellent inherent sensitivity to serial and temporal structure, which make them well adapted for sequence learning [21, 22]. Interestingly, primate cortex is characterized by a vast majority of cortico-cortical connections being local and recurrent [23], and thus cortex is a highly recurrent network [24]. We thus model frontal cortex as a recurrent network. Interestingly, the computational complexity of credit assignment to recurrent connections is high, because it involves keeping track of the role of each connection over successive time steps as the network evolves through its temporal dynamics [21]. One solution is to unwind the recurrent network into a series of feedforward layers where each layer represents the network activation at the next time step. This is efficient [22], but introduces an arbitrary cut off of the recurrent dynamics. In order to allow the recurrent network to maintain complete dynamics, Dominey et al. [25] chose to keep the recurrent connections fixed, with a random distribution of positive and negative connections that ensured a rich network dynamics that represented the influence of new inputs, and the fading effects of previous inputs. The resulting representation of the spatiotemporal structure of the input can then be associated with the desired output function by the modification of simple feedforward connections from the recurrent network to the output neurons. Dominey and colleagues initially proposed that the prefrontal cortex corresponds to the recurrent network, and the striatum, with its dopamine-modifiable corticostriatal connections as the output layer [25]. This was in fact the first characterization of reservoir computing, which was subsequently co-discovered by Jaeger [26, 27], and Maass & Natschlager [28]. It is now well established that frontal cortex can be characterized as a recurrent reservoir model, via demonstrations that the high dimensional representations inherent to these recurrent networks is required for higher cognitive function, and is omnipresent in frontal cortex [19, 29, 30]. The use of the reservoir structure is indeed an originality of our model, and an alternative to classical plastic recurrent networks such as those used to model attractor network dynamics in hippocampus CA3 [31].

We test the hypothesis that the structure of snippet replay from the hippocampus will provide the PFC with constraints that can be integrated in order to contribute to solving the TSP problem. Two principal physical and neurophysiological properties of navigation and replay are exploited by the model and contribute to the system’s ability to converge onto an acceptable solution to the TSP. First, during navigation between baited food wells in the TSP task, non-optimal trajectories by definition cover more distance between rewards than near-optimal ones. Second, during the replay of recently activated places cells, the trajectories are encoded in forward and reverse directions [5, 11]. Exploiting these observations, we test the hypotheses that:

1. With replay biased by distance to reward, non-optimal trajectories will be less represented in replay, allowing the PFC to eliminate non-optimal sub-sequences in constructing the final efficient trajectory.
2. Reverse replay will allow the model to exploit the information provided by a given sequence in forward and backward directions, whereas the actual trajectory run by the rat has one direction only.

In testing these hypotheses, we will illustrate how the system can meet the following challenges:

1. Learn a global place-cell activation sequence from an unordered set of snippets
2. Consolidate multiple non-optimal sequences into a trajectory that efficiently links rewarded locations, thus converging to a good solution to the TSP problem.
3. Experience a trajectory in the forward direction and then learn to generate it in forward and backward direction, including concatenating parts of both forward and reverse replayed snippets in order to generate novel trajectories as demonstrated in Gupta & van der Meer [6]. The objective is to provide a coherent explanation of how critical aspects of replay–notably its modulation by reward, and the forward and reverse aspects, can be exploited by a cortical sequence learning system in order to display novel and efficient navigation trajectory generation.

The model developed in this research provides a possible explanation of mechanisms that allow PFC and hippocampus to interact to perform path optimization. This implies functional connectivity between these two structures. In a recent review of hippocampal–prefrontal interactions in memory-guided behavior Shin and Jadhav [32] outlined a diverse set of direct and indirect connections that allow bi-directional interaction between these structures. Principal direct connections to PFC originate in the ventral and intermediate CA1 regions of the hippocampus [33, 34]. Connections between hippocampus and PFC pass via the medial temporal lobe (subiculum, entorhinal cortex, peri- and post-rhinal cortex) [35], and the nucleus reuniens [36]. Indeed memory replay is observed to be coordinated across hippocampus and multiple cortical areas including V1 [37]. There are direct connections from ventral CA1/subiculum to the rodent medial frontal cortex[38] and from the mFC to dorsal CA1 [39], The connections through the RE nucleus though may be of primary importance for HC-mFC communication [40]. These studies allow us to consider that there are direct and indirect anatomical pathways that justify the modeling of bi-directional interaction between PFC and hippocampus [41].

It is important to note that the model we describe should not be considered to be fully autonomous in driving the behavior of the animal, because it relies on prior experience from which to construct new behavior. This experience is assumed to be generated by visual and olfactive processes that contribute to locally guided behavior.

## Material & methods

Experiments are performed on navigation trajectories (observed from rat behavior, or generated automatically) that represent the recent experience from the simulated rat. Snippets are extracted from this experience, and used to train the output connections of the PFC reservoir. This requires the specification of a model of place-cell activation in order to generate snippets. Based on this training, the sequence generation performance is evaluated to test the hypotheses specified. The evaluation requires a method for comparing sequences generated with expected sequences that is based on the Fréchet distance.

### Navigation behavior and trajectories

A trajectory is a sequence of N contiguous two-dimensional coordinates sampled from time *t*_1_ to time *t*_*N*_ noted *L*(*t*_1_→*t*_*N*_) that corresponds to the rat’s traversal of the baited feeders. The spatial resolution of trajectories are depicted at 20 *points*/*m* along the trajectory. Experiments were performed using navigation trajectories, including those displayed in Fig 1, based on data recorded from rats as they ran the TSP task [13] in a circular arena having a radius of 151cm. Twenty-one fixed feeders are distributed according to a spiral shape. In a typical configuration, five feeders are baited with a food pellet. For a given configuration, the rat runs several trials which are initially random and inefficient, and become increasingly efficient over successive trials, characterizing the TSP behavior [13]. Rat data that characterizes the TSP behavior is detailed in S1 Text, section Rat navigation data. The principle concept is that TSP behavior can be characterized as illustrated in Fig 1, where a system that is exposed to trajectories that contain elements of the efficient path can extract and concatenate these sub-sequences in order to generate the efficient trajectory.

### Place-cells

The modeled rat navigates in a closed space of 2x2 meters where it can move freely in all direction within a limited range (± 110° left and right of straight ahead), and encodes locations using hippocampus place-cell activity. A given location *s* = (*x*,*y*) is associated with a place-cell activation pattern by a set of 2D Gaussian place-fields:

$$f_{k}(s)=e^{-\frac{{\left\|s-c_{k}\right\|}^{2}}{w_{k}}}$$ (1)

Where:

- *k* is the index of the place-cell
- *f*_*k*_(*s*) is the mean firing rate of the *k*^*th*^ place-cell
- *c*_*k*_ is the (*x*,*y*) coordinate of the *k*^*th*^ place-cell

- $w_{k}=\frac{r_{k}^{2}}{-\log\left(\Theta \right)}$ is a constant that will constrain the highest activations of the place-cell to be mostly contained in a circle of radius *r*_*k*_, centered in *c*_*k*_
- *r*_*k*_ is the radius of the *k*^*th*^ place-field
- *Θ* is the radius threshold which controls the spatial selectivity of the place-cell

**Fig 2:**
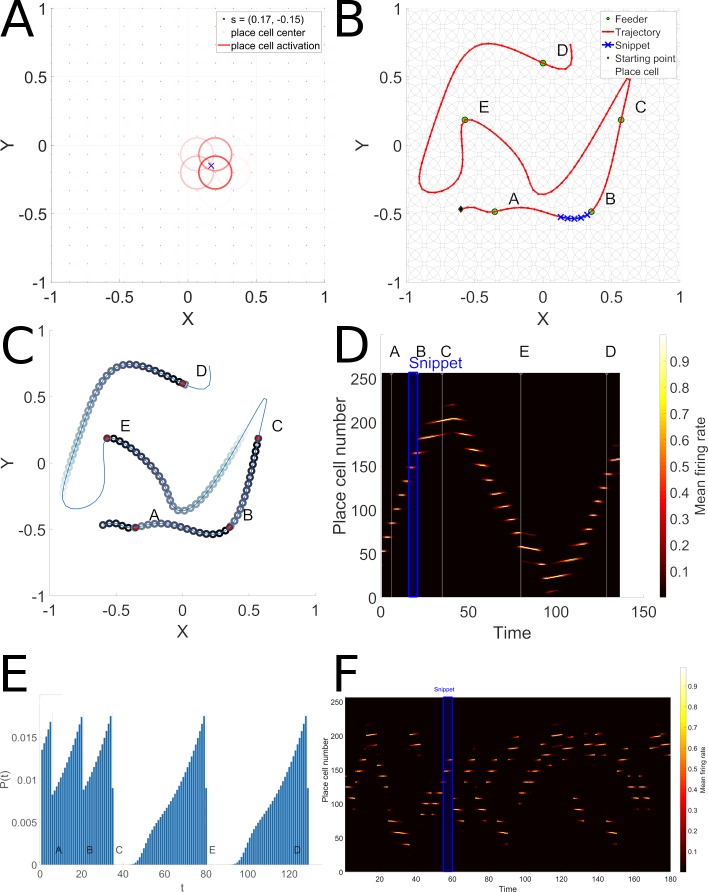
Place-cell and snippet coding. Panel A represents the place-cell activations that correspond to a single point. Place-cell centers are represented by red points and the mean firing rate of each place-cell by a red circle with a fixed radius, centered on the place-cell center. The transparency level of the circle represents the magnitude of the mean firing rate. Panel B depicts the ABCED trajectory, and a snippet randomly drawn along the trajectory. The snippet length is *s* = 5. Panel C represents the snippet replay likelihood as learnt by the Hippocampal replay model by propagation of reward from rewarded locations at ABCE and D. Panel D represents the raster of the place-cell activation along the ABCED trajectory. The time index where feeders A,B,C,D and E are encountered during the ABCED trajectory are tagged above the raster and represented by a thin white vertical line. The snippet represented in panel B is emphasized by a blue rectangle in panel D. Panel E represents the spatial extent of the snippet replay likelihood. F illustrates part of a typical random replay episode, where multiple snippets from remote locations are replayed.

Parameter *w*_*k*_ is a manner of defining the variance of the 2D Gaussian surface with a distance to center related parameter *r*_*k*_. We model a uniform grid of 16x16 Gaussian place-fields of equal size (mimicking dorsal hippocampus). In Fig 2 the spatial position and extent of the place fields of several place-cells is represented in panel A by red circles. The degree of red transparency represents the mean firing rate.

A mean firing rate close to one will result in a bright circle if the location *s* is close to the place-field center *c*_*k*_ of the place-cell *k*. For a more distant place-field center *c*_*l*_ of place-cell *l*, the mean firing rate will be less important and the red circle representing this mean firing rate will be dimmer.

Thus, at each time step the place-cell coding that corresponds to a particular point in a trajectory is defined as the projection of this *L*(*t*_*n*_) point through *K* radial basis functions (i.e. Gaussian place-fields spatial response)

$$X_{in}(t_{n})={\left\{f_{k}(L(t_{n}))\right\}}_{k\in 1\ldots K}$$ (2)

Each coordinate of the input vector *X*_*in*_(*t*_*n*_) represents the mean firing rate of hippocampus place-cells and its value lies between 0 and 1. Fig 2 represents in panel B the ABCED trajectory *L*(*t*_1_→*t*_*N*_) and the corresponding place-cell mean firing rate raster *X*_*in*_(*t*_1_→*t*_*N*_) is depicted in panel C.

### Hippocampus replay

The hippocampus replay observed during SWR complexes in the active rest phase (between two trials in a given configuration of baited food wells) is modeled by generating condensed (time compressed) sub-sequences of place-cell activation patterns (snippets) that are then replayed at random so as to constitute a training set. The sampling distribution for drawing a random place-cell activation pattern might be uniform or modulated by new or rewarding experience as described in [1]. Ambrose and Pfeiffer [9] demonstrated that during SWR sequences place-cell activation occur in reverse order at the end of a run. We define a random replay generative model that learns to preferentially generate snippets associated to a reward by using reverse replay. For a trajectory encountered in forward direction, reverse replay allows the model to anticipate the reward by propagating the reward information in the backwards direction. Once learnt the model is able to generate snippets in forward and reverse order, hence representing parts of a trajectory in reverse or forward direction. This innovative method for spatial propagation of reward during replay yields a computationally simple form of reinforcement learning.

We define a snippet as the concatenation of a pattern of successive place-cell activations from a previously traversed trajectory:

$$S(n;s)=X_{in}(t_{n}\to t_{n+s})$$ (3)

Where: *s* is the number of place-cell activations (or the snippet length), and *n* is the offset in the trajectory. Replay occurs during SWRs at ~150-200Hz with a duration ~50–120 ms [2], so snippet length *s* in our experiments is typically 10 and varies from 5–20. We define a replay episode as the period between trials in the TSP experiment (on the order of 2–5 minutes) during which replay occurs. The duration of a replay episode is constrained by a time budget *T*, defined in simulation update cycles. Place cell activations in the simulated replay occur on each time step, with each time step corresponding to 5ms, or a 200Hz update rate. A replay episode *E* is a set of snippets of length *s*:

E(s)={S(n;s)} (4)

such that sum of the durations of snippets replayed in *E* is constrained to be ≤ *T*. In a typical experiment described below T = 10000 and s = 10, which corresponds to 1000 snippets of length 10. In order to respect ecological orders of magnitude, we consider that during 2–5 minutes of intertrial delay in the TSP task, SWRs occur at ~1Hz, corresponding to ~120–300 SWRs. In a given SWR it is likely that across the dorsal hippocampus, 100s - 1000s of places cells will fire, corresponding to an order of 10^1^–10^2^ snippets per SWR. This parallel replay of multiple independent snippets within an SWR is hypothesized but has not yet been experimentally observed. Over 2–5 minutes, this corresponds to a lower bound of 1.2x103—and an upper bound of 3x10^4^ snippets during the intertrial period. We conservatively model this at 1x10^3^. In a given episode snippet length *s* is fixed. Individual snippets are spatially coherent, while successive snippets are not, and can start from random locations along previously experienced trajectories.

In Fig 2, Panel B represents a particular trajectory through feeders A, B, C, E and D. The depicted snippet is a sub-sequence of 5 contiguous locations belonging to the ABCED sequence. The B and E feeders are baited and marked as rewarding (R_1_ and R_2_). Panel B shows the spatial extent of a given snippet chosen in sequence ABCED and panel C shows the place-cell activation pattern of the ABCED trajectory and the corresponding snippet location in the raster.

#### Reward propagation

The snippet replay model favors snippets that are on efficient paths linking rewarded sites (e.g. paths linking feeders A, B and C in panel B Fig 2), and not those that are on inefficient paths (as in paths linking C, D and E in the same panel). This is achieved by propagating reward value backwards from rewarded locations, and calculating the probability of replay as a function of proximity to a reward. Panel D of Fig 2 illustrates the resulting probability distributions for snippet selection along the complete path. Panel E represents the spatial extent of snippet replay likelihood. Note that the paths linking A, B and C have the highest probabilities for snippet replay.

Hippocampus place-cell replay can occur in forward or backward direction as suggested in [11]. We model the reverse replay as follows: For a given trajectory of *N* samples, there are *N*−*s* possible snippets that can be replayed but only a limited number of snippets will be selected to fit the time budget *T*. A snippet *S*(*n*;*s*) has a likelihood of being replayed if it is related to a reward prediction. A generative model of snippet replay likelihood P is computed by first defining the reward prediction V over time indices sequence *t*_1_−>*t*_*N*_ of a trajectory described by N samples, noted *V*(*t*_1_−>*t*_*N*_). This reward prediction value corresponds to the replay likelihood illustrated in Fig 2C and 2E. It is initialized to a small positive random value as described in the algorithm replay-initialize:

**Table:** 

Replay-initialize		
**Input**		
	**ϵ**, a small positive constant	
**Output**		
	V, the time indexed reward prediction	
**Algorithm**		
**1**	**for all** i∈{1,..,N} **do**	/* For all time indices i in V*/
**2**	***V***(***i***)**←random**(**U**[**0,ϵ**])	/* Initialize V(i) by drawing a small positive random number from a uniform distribution*/
**3**	**end for**	
**4**	**return** V	

Replay-initialize algorithm pseudocode

Then the reward prediction V is learned by propagating a time delayed reward information according to the replay direction (forward/reverse) and the snippet duration. This is described in the *replay-learn algorithm* pseudocode.

**Table:** 

Replay learn		
**Input**		
	R, the time indexed instantaneous reward	
	V, the time indexed reward prediction	
	*β*_*learn*_, the reverse rate used during learning	
	*α*, the learning rate	
	*γ*, the discount rate	
	T, the time budget allocated to the replaylearn algorithm	
**Output**		
	V, the time indexed reward prediction	
**Algorithm**		
**1**	**while** T > 0 **do**	
**2**	**for all** i∈{1,..,*N*} **do**	/* For all N time indices i in V */
**3**	$P(i)\leftarrow \frac{V(i)}{\sum_{j=1}^{N}V(j)}$	/* Compute the snippet replay likelihood by normalizing the reward prediction V*/
**4**	**end for**	
**5**	t←random(P)	/* Select a random time index t by sampling from P distribution */
**6**	r←random(U[0,1])	/* Draw a random number r from the uniform distribution U[0, 1] */
**7**	**if** *r*<B_*learn*_ **then**	
**8**	*τ*←{*t*,*t*−1,…,max(1,*t*−*s*+1)}	/* Define a snippet τ in reverse direction */
**9**	**else**	
**10**	*τ*←{*t*,*t*+1,…,min(*N*,*t*+*s*)}	/* Define a snippet τ in forward direction */
**11**	**end if**	
**12**	**for** i **in** 2…*length*(*τ*) **do**	/* For all time indices i in snippet τ */
**13**	*V*(*τ*_*i*_)←*α*(*R*(*τ*_*i*−1_)+*γV*(*τ*_*i*−1_))+(1−*α*)*V*(*τ*_*i*_)	/* Apply a TD−λ algorithm on V by using successive time indices contained in τ)*/
**14**	**end for**	
**15**	*T*←*T*−*length*(*τ*)	/* compute the remaining time budget*/
**16**	**end while**	
**17**	**return** V	

Replay-learn algorithm pseudocode

The timespan and the direction of a snippet acts as a propagation vector during the estimation phase of the snippet replay likelihood. Line 13 of the **replay-learn** algorithm shows that V is updated as a convex combination of the current estimate of the reward information *V*(*τ*_*i*_) at the next time step and the instantaneous reward information *R*(*τ*_*i*−1_)+*γV*(*τ*_*i*−1_) based on the previously observed reward signal *R*(*τ*_*i*−1_) and delayed previous reward estimate *γV*(*τ*_*i*−1_)). It implements a form of temporal difference learning. It is sufficient to define a coarse reward signal as:

$$R(t)=\left\{ \begin{array}{c} 1\;if\;a\;baited\;feeder\;is\;encountered\;at\;time\;t\\ 0\;otherwise \end{array}\right.$$

The results of this reward propagation is visualized in Fig 2C and 2E. Once the reward prediction is learned, it is used to generate snippets to train the PFC model. Note that this implies a reward prediction estimation phase described above, and a snippet generation phase, described below. For clarity we separate these phases, but they can be combined, by using the snippets generated in the estimation phase for training the PFC.

#### Snippet generation during replay

The **replay-generate** algorithm is applied to the condensed version of the place-cell activation sequence that was previously encountered. This will result in the extraction of a set of snippets biased by the reward propagation. This set of snippets is a replay episode which models the place cell-activation observed during SWR in the inter-trial interval.

**Table:** 

Replay-generate		
**Input**		
	V, the time indexed reward prediction	
	*β*_*generate*_, the reverse rate used during snippet generation	
	T, the time budget allocated to the replay-generate algorithm	
**Output**		
	S the collection of generated snippets	
**Algorithm**		
**1**	**for all** i∈{1,..,N_k_} **do**	/* For all N time indices i in V */
**2**	$P(i)\leftarrow \frac{V(i)}{\sum_{j=1}^{N}V(j)}$	/* Compute the snippet replay likelihood P by normalizing the reward prediction V*/
**3**	**end for**	
**6**	*S*←∅	/* Initialize S as an empty collection */
**9**	**while** T > 0 **do**	
**12**	t←random(P)	/* Select a random time index t by sampling from P distribution */
**13**	r←random(U[0,1])	/* Draw a random number r from the uniform distribution U[0, 1] */
**14**	**if** *r*<B_*generate*_ **then**	
**15**	*τ*←{*t*,*t*−1,…,max(1,*t*−*s*+1)}	/* Define a snippet τ in reverse direction */
**16**	**else**	
**17**	*τ*←{*t*,*t*+1,…,min(*N*,*t*+*s*)}	/* Define a snippet τ in forward direction */
**18**	**end if**	
**19**	*T*←*T*−*length*(*τ*)	/* compute the remaining time budget*/
**22**	*S*←*S*∪*τ*	/* append snippet τ in S*/
**23**	**end while**	
**24**	**return** S	

Replay-generate algorithm pseudocode

As indicated above, during a 2 minute (120 second) intertrial interval, we can assume that ~100 SWRs will occur, with ~10 cell sequences firing per SWRs across all the place cells with fields on the maze, yielding ~1000 snippets per replay episode. Thus, learning with 1000 snippets can be considered to take place in a biologically realistic timeframe. New in vivo imaging techniques after spatial navigation in such a multi goal tasks would be useful in refining these numbers.

These snippets will serve as inputs to the reservoir model of PFC described below. As illustrated in Fig 2D and 2E, the replay is biased by proximity to reward, which has been spatially propagated. Based on this reward propagation, the behavior generated by the model favors the shortest trajectories that cover the learned baited feeders. The propagation of reward is illustrated in Fig 2C and 2E. Panel C displays the 2D propagation of reward value backwards from the rewarded targets. Note that for short trajectories between rewards (e.g. sub-sequence ABC) there is a continuous dense reward distribution that will favor replay of this sub-sequence. For long meandering trajectories the reward density diminishes to zero (e.g. sub-sequence CE), thus disfavoring replay of snippets on these sub-sequences. Panel 2E displays the 1D equivalent view of the reward propagation. Again note the continuous high density of reward along ABC, vs. the discontinuities with zero reward values for the sub-sequences CED. Starting from feeders C, D or E, reward prediction is discontinuous and/or absent (replay likelihood is zero in areas along these segments) thus the PFC model will fail to consolidate a path along these segments.

### Reservoir model of PFC for snippet consolidation

We model the prefrontal cortex as a recurrent reservoir network. Reservoir computing refers to a class of recurrent network models with fixed recurrent connections. The reservoir units are driven by external inputs and the network dynamics provides a high dimensional representation of the inputs from which the desired outputs can then be read out by a trained linear combination of the reservoir unit activities. The principle has been co-developed in distinct contexts as the temporal recurrent network [20], the liquid state machine [28], and the echo state network [26]. The version that we use to model the frontal cortex employs leaky integrator neurons in the recurrent network. This model of PFC is particularly appropriate because the recurrent network generates dynamic state trajectories that will allow overlapping snippets to have overlapping state trajectories. This property will favor consolidation of a whole sequence from its snippet parts. At each time-step, the network is updated according to the following schema:

The hippocampus place-cells project into the reservoir through feed-forward synaptic connections noted *W*_*ffwd*_. The projection operation is a simple matrix-vector product. Hence, the input projection through feed-forward synaptic connections is defined by:

$$U_{ffwd}(t_{n})=W_{ffwd}*X_{in}(t_{n})$$ (5)

Where:

- *W*_*ffwd*_ is a fixed connectivity matrix whose values do not depend on time.

Synaptic weights are randomly selected at the beginning of the simulation. Practically speaking [42], sampling U[−1,1] a uniform distribution is sufficient. A positive synaptic weight in a connectivity matrix models an excitatory connection and a negative weight models an inhibitory connection between two neurons (that could be implemented via an intervening inhibitory interneuron). Let N be the number of neurons in the reservoir. Reservoir's neurons are driven by both sensory position inputs *X*_*in*_(*t*_*n*_) and, importantly by the recurrent connections that project an image of the previous reservoir state back into the reservoir. The recurrent projection is defined as:

$$U_{rec}(t_{n})=W_{rec}*X_{res}(t_{n-1})$$ (6)

Where:

- *W*_*rec*_ is a N by N square connectivity matrix.
- *X*_*res*_ is the reservoir activation (mean firing rate) (Eq (10)

Synaptic weights are drawn from a U[−1,1] uniform distribution, scaled by a $S(N;K)=K\frac{1}{\sqrt{N}}$ factor. The same sign convention as in Eq (5) applies for the recurrent connectivity matrix.

Self-connections (i.e. $w_{rec}^{i,i}$ with *i*∈1…*N*) are forced to zero. *W*_*rec*_ is also fixed and its values do not depend on time. The contributions of afferent neurons to the reservoir’s neurons is summarized by

$$U_{res}(t_{n})=U_{ffwd}(t_{n})+U_{rec}(t_{n})$$ (7)

The membrane potential of the reservoir’s neurons *P*_*res*_ then is computed by solving the following ordinary derivative equation (ODE):

$$\tau \frac{\partial P_{res}}{\partial t}=-P_{res}(t_{n-1})+U_{res}(t_{n})$$ (8)

Where:

- *τ* is the neuron’s time constant. It models the resistive and capacitive properties of the neuron’s membrane.

In this article, we will consider a contiguous assembly of neurons that share the same time constant. The inverse of the time constant is called the leak rate and is noted *h*. By choosing the Euler’s forward method for solving Eq (8), the membrane potential is computed recursively by the equation:

$$P_{res}(t_{n})=h*U_{res}(t_{n})+{(1-h)*P}_{res}(t_{n-1})$$ (9)

This is a convex combination between instantaneous contributions of afferents neurons *U*_*res*_(*t*_*n*_) and the previous value *P*_*res*_(*t*_*n*−1_) of the membrane potential. The current membrane potential state carries information about the previous activation values of the reservoir, provided by the recurrent weights. The influence of the history is partially controlled by the leak rate. A high leak rate will result in a responsive reservoir with a very limited temporal memory. A low leak rate will result in a slowly varying network whose activation values depend more on the global temporal structure of the input sequence.

Finally, the mean firing rate of a reservoir’s neuron is given by:

$$X_{res}(t_{n})=\sigma_{res}(P_{res}(t_{n});\Theta_{res})$$ (10)

Where:

- σ_*res*_ is the non-linear activation function of the reservoir neurons
- *Θ*_*res*_ is a bias that will act as a threshold for the neuron’s activation function.

We chose a σ_*res*_≡*tanh* hyperbolic tangent activation function with a zero bias for *Θ*_*res*_. Negative firing rate values represent the inhibitory/excitatory connection type in conjunction with the sign of the synaptic weight. Only the product of the mean firing rate of the afferent neuron by its associated synaptic weight is viewed by the leaky integrator neuron. See S1 Text High dimensional processing in the reservoir for more details on interpreting activity in the reservoir.

#### Learning in modifiable PFC connections to readout

**Fig 3:**
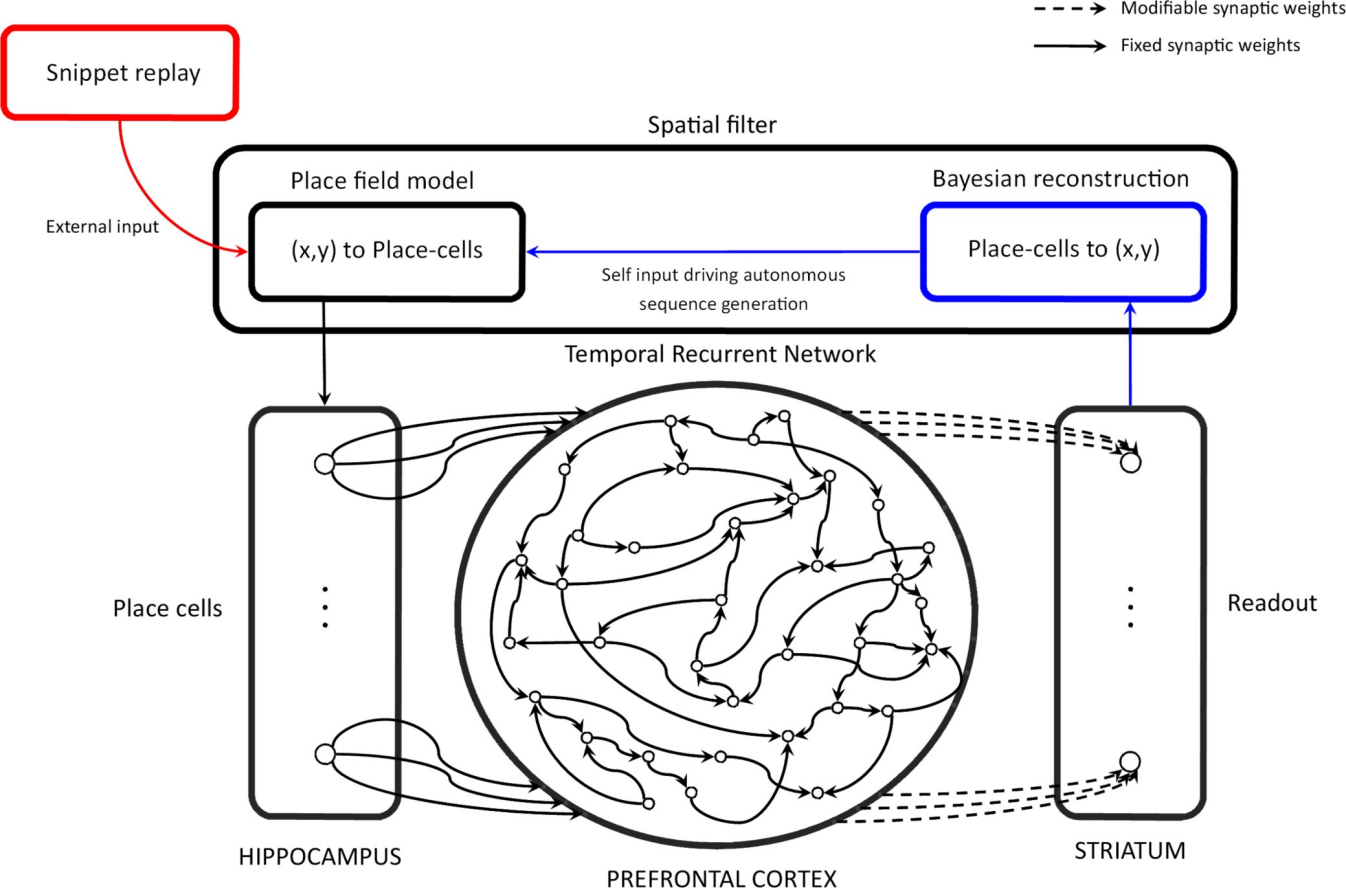
Reservoir computing model. The Temporal Recurrent Network (TRN) is a model of the prefrontal cortex (PFC) that takes into account cortico-cortical loops by defining a fixed recurrent adjacency matrix for the leaky integrator neurons that model PFC neurons. Inputs of the TRN are modelled hippocampus (HIPP) place-cells. During the training phase, place-cells activations are provided by the algorithmic model of SWR replay (red pathway), and the striatum model learns to predict the next place-cell activation from the PFC model states by modifying the synaptic weights that project the PFC model into the striatum model according to the delta learning rule. During the generation phase, the model is no longer learning and the place-cell activation patterns result from the new position of the agent, reconstructed with a Bayesian algorithm from the next place-cell activation prediction of the modeled striatum (blue pathway).

Based on the rich activity patterns in the reservoir, it is possible to decode the reservoir's state in a supervised manner in order to produce the desired output as a function of the input sequence. This decoding is provided by the readout layer and the matrix of modifiable synaptic weights linking the reservoir to the readout layer, noted *W*_*ro*_ and represented by dash lines in Fig 3.

The readout activation pattern *X*_*ro*_(*t*_*n*_) is given by the equation:

$$X_{ro}(t_{n})={\sigma_{ro}(W}_{ro}*X_{res}(t_{n});\Theta_{ro})$$ (11)

Where:

- σ_*ro*_ is the non-linear activation function of the readout neurons
- *Θ*_*ro*_ is a bias that will act as a threshold for the neuron’s activation function

We chose a σ_*ro*_≡*tanh* hyperbolic tangent activation function with a zero bias for *Θ*_*ro*_.

Notice that the update algorithm described above is a very particular procedure inherited from feedforward neural networks. We chose to use it because it is computationally efficient and deterministic.

Once the neural network states are updated, the readout synaptic weights are updated by using a stochastic gradient descent algorithm. By deriving the Widrow-Hoff Delta rule [43] for hyperbolic tangent readout neurons, we have the following update equation:

$$W_{ro}(t_{n})=W_{ro}(t_{n-b+1})+\alpha *X_{res}(t_{n-b+1}\to t_{n})*(X_{ro}(t_{n-b+1}\to t_{n})-X_{des}(t_{n-b+1}\to t_{n}))*(1-X_{ro}{\left(t_{n-b+1}\to t_{n}\right)}^{2})$$ (12)

Where:

- *α* is a small positive constant called the learning rate
- *t*_*n*−*b*+1_→*t*_*n*_ is the concatenation of b time steps from *t*_*n*−*b*+1_ to *t*_*n*_

When *b* = 1, Eq (12) computes a stochastic gradient descent. The case when *b*>1 is called a mini-batch gradient descent and allows one to estimate the synaptic weight gradient base on b successive observations of predicted and desired activation values. A mini batch gradient allows one to compute efficiently and robustly the synaptic weight gradient. Empirically, *b* = 32 gives satisfying results.

In this study, we will focus on the prediction of the next place-cell activation pattern (*X*_*des*_):

$$X_{des}(t_{n})=X_{in}(t_{n+1})$$ (13)

This readout is considered to take place in the striatum, as part of a cortico-striatal learning system. This is consistent with data indicating that while hippocampus codes future paths, the striatum codes actual location [44].

#### Training

The model is trained using a dataset, typically 1000 snippets, that is generated by the snippet replay mechanism described above in the paragraph on Hippocampus replay. The snippet dataset is generated from trajectories that were run in previous trials. In the consolidation experiments, the previous trials correspond to the trajectories illustrated in Fig 1B–1D. The readout synaptic weights are learned online by using the learning rule described in the Learning in Modifiable PFC Connections to Readout section. The model does not receive any form of feedback from the environment and it learns place-cell activation sequences based only on random replay of snippets.

Between each sequence of the training set (snippets in our case), the states of the reservoir and readout are set to a small random uniform value centered on zero. This models a time between the replays of two snippets that is sufficiently long for inducing states in the neural network that are not correlated with the previous stimulus. This is required for having the same effect as simulating a longer time after each snippet but without having to pay the computational cost associated to this extra simulation time.

### Embodied simulation of sensory-motor loop via the spatial filter

Once the model is trained, we need to evaluate its performance and the trajectories it can generate. The model is primed with the first *p* steps of the place-cell activation sequence the model is supposed to produce. This sequence is called the target sequence. Then the model’s ability to generate a place-cell activation sequence is evaluated by injecting the output prediction of the next place-cell activation pattern as the input at the next step. In this iterative procedure, the system should autonomously reproduce the trained sequence pattern of place-cell activations.

Predicted place-cell activation values might be noisy, and the reinjection of even small amounts of noise in this autonomous generation procedure can lead to divergence. We thus employ a procedure that determines the location coded by the place-cell activation vector output, and reconstructs a proper place-cell activation vector coding this location. We call this denoising procedure the spatial filter as referred to in Fig 3.

We model the rat action as ‘reaching the most probable nearby location’. Since only the prediction of the next place-cell activation pattern *η* is available, we need to estimate the most probable point s*(*t*_*n*+1_) = (x*(*t*_*n*+1_),y*(*t*_*n*+1_)). From a Bayesian point of view, we need to determine the most probable next location *s*(*t*_*n*+1_), given the current location *s*(*t*_*n*_) and the predicted place-cell activation pattern *η*(*t*_*n*_). We can state our problem as:

$$s^{*}(t_{n+1})=argmax_{s(t_{n+1})}P(s(t_{n+1})|\eta (t_{n}),s(t_{n}))+u$$ (14)

Where:

- *u* is a noise function sampling a uniform distribution U(0,m)

*u* is useful at least in degenerate cases when a zero place-cell activation prediction generates an invalid location coding. It is also used for biasing the generation procedure and to explore other branches of the possible trajectories the model can generate as described in section Evaluating Behavior with Random walk.

The system is then moved to this new location s* and a new noise/interference free place-cell activation pattern is generated by the place-field model. We refer to this place-cell prediction/de-noising method as the spatial filter, which emulates a sensory-motor loop for the navigating rat in this study. Fig 3 depicts this sensory motor loop.

### Evaluating behavior with random walk

**Fig 4:**
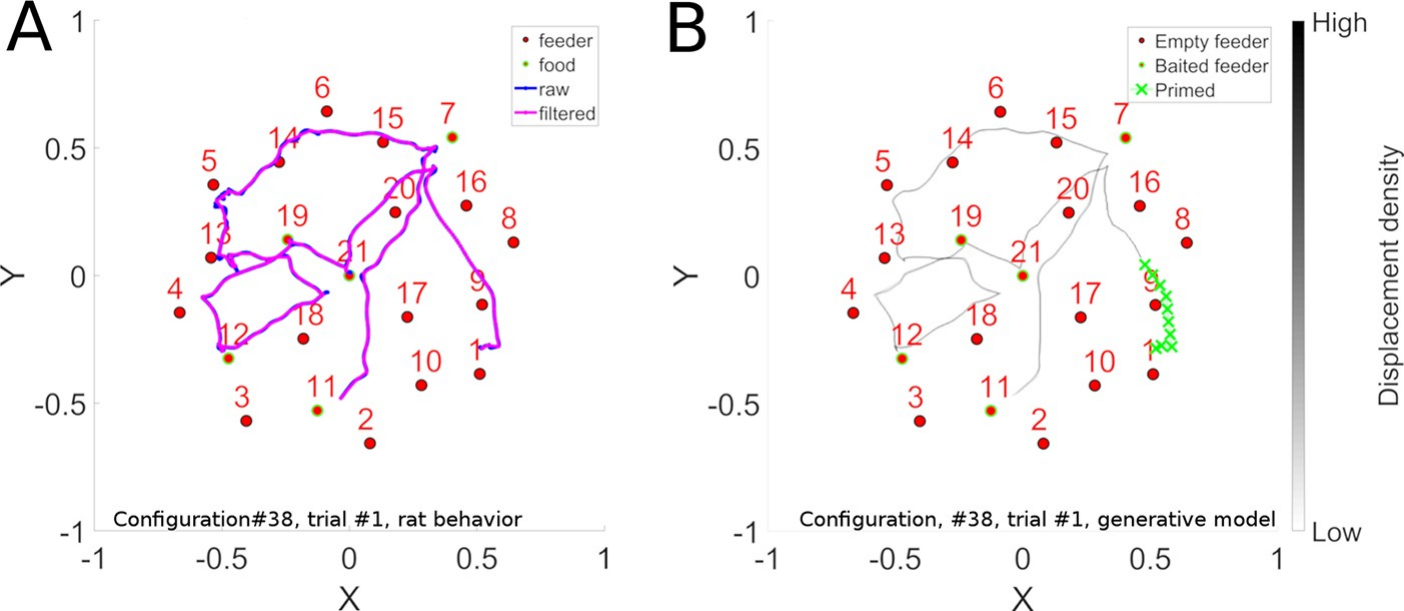
Sequence learning. Panel A illustrates a long convoluted trajectory taken by a rat in configuration 38. Panel B illustrates the probability maps of trajectories generated by the trained model in autonomous sequence generation mode. The 2D trajectory histogram is generated by superposing the trajectories generated when 10 batches of 100 reservoirs each were trained and each model instance was evaluated 10 times with noise. Note that there are two locations where the trajectory crosses itself. At the point of crossing, there are two possible paths that preserve path continuity. The system has memory of the context of how it got to that intersection point, and thus can continue on that trajectory. This illustrates that the model is well able to learn such complex sequences.

Once the model has been trained, it is then primed with place-cell activation inputs corresponding to the first few steps of the trajectory to be generated. The readout from the PFC reservoir generates the next place-cell activation pattern in the trajectory, which is then reinjected into the reservoir via the spatial filter, in a closed loop process. This loop evaluation procedure is called *autonomous* generation. In order to evaluate the model in a particular experimental condition, several instances of the same model are evaluated multiple times in a random walk procedure. The batch of generated trajectories (typically 1000) are accumulated in a stencil buffer which acts as a two dimensional histogram showing the most frequently generated trajectories. The arena is drawn with its feeders and a vector field is computed from trajectories in order to show the main direction of these trajectories. Trajectories are superimposed and summed, resulting in a two-dimensional histogram representing the space occupied by trajectories. Fig 4 shows an example of random walk trajectories, illustrating the model’s ability to autonomously generate a long and complex sequence when learning without snippets. Fig 4B illustrates a 2D histogram formed by superimposing trajectories autonomously generated by 1000 reservoirs evaluated ten times each with noise, in order to validate the robustness of the behavior.

In cases where small errors in the readout are reinjected as input, they can be amplified, causing the trajectory to diverge. It is possible to overcome this difficulty by providing as input the expected position at each time step instead of the predicted position. The error/distance measurement can still be made, and will quantify the diverging prediction, while allowing the trajectory generation to continue. This method is called *non-autonomous* generation and it evaluates only the ability of a model to predict the next place-cell activation pattern, given an input sequence of place-cell activations.

### Comparing produced and ideal sequences using discrete Fréchet distance

The joint PFC-HIPP model can be evaluated by comparing an expected place-cell firing pattern with its prediction by the readout layer. At each time step, an error metric is computed and then averaged over the duration of the expected neurons firing rate sequence. The simplest measure is the mean square error. This is the error that the learning rule described in Eq (12) minimizes.

Although the model output is place-cell coding, what is of interest is the corresponding spatial trajectory. A useful measurement in the context of comparing spatial trajectories is the discrete Fréchet distance. It is a measure of similarity between two curves that takes into account the location and ordering of the points along the curve. We use the discrete Fréchet distance applied to polygonal curves as initially described in Eiter and Mannila [45]. In [46] the Discrete Fréchet distance *F* between two curves *A* and *B* is defined by:

$$F(A,B)=\underset{\alpha ,\beta }{\min}\underset{t\in [1,m+n]}{\max}\left[d(A(\alpha (t)),B(\beta (t)))\right]$$ (15)

Where *d*(.,.) is the Euclidean distance, *m* is the number of steps of the curve A, *n* is the number of steps of the curve B, and *α*,*β* are reparametrizations of the curves A and B. Parameterization of this measure is described in more detail in S1 Text Frechet distance parameters.

## Results

For robustness purposes, results are based on a population of neural networks rather than a single instance. The population size is usually 1000 for evaluating a condition and the metrics described above are aggregated by computing their mean *μ*(.) and standard-deviation *σ*(.). For convenience, we define a custom score function associated to a batch of coherent measurements as:

score(X)=μ(X)+σ(X) (16)

Results having a low mean and standard deviation will be reported as low score whilst other possible configurations will result in a higher score. We chose this method rather than Z-score, which penalizes low standard deviations. We first established that the model displays standard sequence learning capabilities (e.g. illustrated in Fig 4) and studied parameter sensitivity (see S1 Text Basic Sequence learning and parameter search), and then addressed consolidation from replay.

### Consolidation from snippet replay

The model is able to learn and generate navigation sequences from place-cell activation patterns. The important questions is whether a sequence can be learned by the same model when it is trained on randomly presented snippets, instead of the continuous sequence.

**Fig 5:**
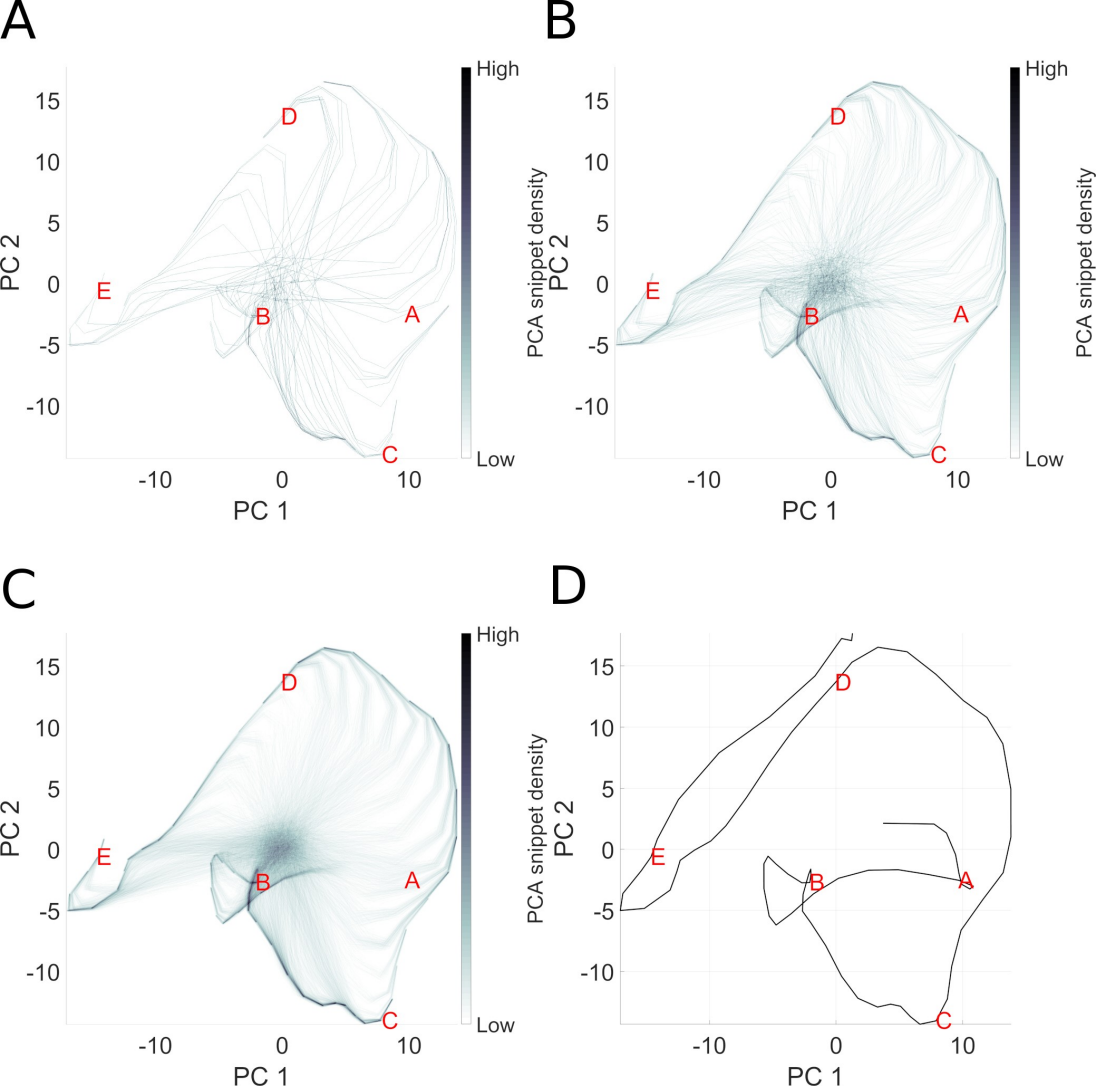
Illustration of snippet integration in reservoir state space. Here we visualize the high dimensional reservoir space in a low (2D) PCA space, in order to see how pieces (snippets) of the overall sequence are consolidated. In this experiment, the sequence ABCDE is broken into snippets, which are then used to train the model. The challenge is that only local structure is presented to the model, which must consolidate the global structure. Panels A-C represent the state trajectory of reservoir activation after 100, 1000 and 10000 snippets. While each snippet represents part of the actual trajectory, each is taken out of its overall spatial context in the sequence. Panel D represents the trajectory of reservoir state during the complete presentation of the intact sequence. Panel C reproduces this trajectory, but in addition we see “ghost” trajectories leading to the ABCDE trajectory. These ghost elements represent the reservoir state transitions from an initial random state as the first few elements of each snippet take the reservoir from the initial undefined state onto the component of the ABCDE trajectory coded by that snippet.

In this experiment, no reward is used, and thus each snippet has equal chance of being replayed. The only free parameter is the snippet size. In order to analyze the reservoir response, we collect the state-trajectories of reservoir neurons when exposed to snippets. Recall that the internal state of the reservoir is driven by the external inputs, and by recurrent internal dynamics, thus the reservoir adopts a dynamical state-trajectory when presented with an input sequence. Such a trajectory is visualized in Fig 5D. This is a 2D (low dimensional) visualization, via PCA, of the high dimensional state transitions realized by the 1000 neurons reservoir as the input sequence corresponding to ABCDE is presented. Panels A-C illustrate the trajectories that the reservoir state traverses as it is exposed to an increasing number of randomly selected snippets generated for the same ABCDE sequence. We observe that as snippets are presented, the corresponding reservoir state-trajectories start roughly from the same point because of the random initial state of the reservoir before each snippet is replayed. Then the trajectories evolve and partially overlap with the state-trajectory produced by the complete sequence. In other words, snippets quickly drive the reservoir state from an initial random activation (corresponding to the grey area at the center of each panel) onto their corresponding locations in the reservoir activation state-trajectory of the complete sequence. Replaying snippets at random allows the reservoir to reconstruct the original intact reservoir state trajectory because the reservoir states overlap when snippet trajectories overlap.

Thus, we see that the state trajectories traversed by driving the reservoir with snippets overlaps with those from the original intact sequence. As illustrated in 5A and 5B, 100 to 1000 snippets are required for allowing the consolidation to occur in the readout layer with the learning rule described in Eq (12). A smaller learning error is achieved with 10000 snippets because the reservoir states that correspond to the whole sequence depicted in panel D are observed more often and the error gradient corrected more often by modifying the readout synaptic weights. See further details of sequence learning by snippet replay in S1 Text Sequence complexity effects on consolidation.

### Longer paths are rejected

**Fig 6:**
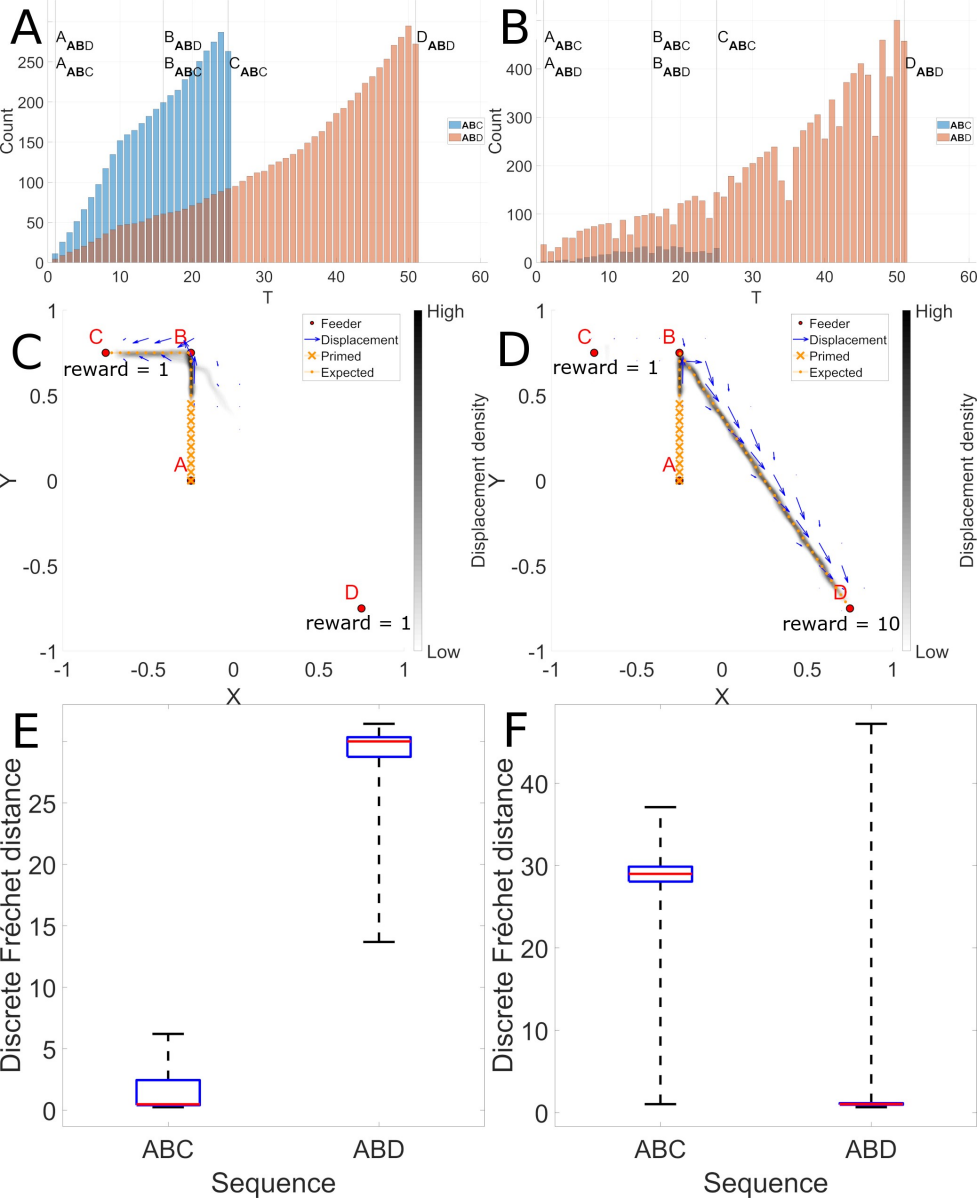
Longer paths are rejected (left), and stronger rewards are favored (right). Panels A and B illustrate snippet counts for T maze trajectories pictured in panels C and D. In Panel C, sequences begin at location A, and rewards are given at locations C and D. Based on the reward proximity and propagation, there is a higher probability of snippets being selected along path AC than path AD. This is revealed in panel A, a histogram of snippets for the sequences ABC (in Blue) and ABD (in Orange). Panels B and D illustrate how distance and reward intensity interact. By increasing the strength of the reward, a longer trajectory can be rendered virtually shorter and more favored, by increasing the probability that snippets will be selected from this trajectory, as revealed in Panel B. Panels C and D reveal the 2D trajectory histograms generated by superposing the trajectories generated when 10 batches of 100 reservoirs each were trained and each model instance was evaluated 10 times with noise. Panel E and F confirm a robust tendency to generate autonomously sequences significantly similar to the ABC and ABD sequence respectively (p-value = 0).

Here we examine how using reward proximity to modulate snippet replay probability distributions (as described in the hippocampal replay description) allows the rejection of longer, inefficient paths between rewarded targets. In this experiment, 1000 copies of the model are run 10 times. Each is exposed to the reward modulated replay of two sequences ABC and ABD having a common prefix AB as illustrated in Fig 6. The model is trained on snippets replayed from trajectories ABC and ABD. The random replay is not uniform and takes into account the reward associated with a baited feeder when food was consumed, as describe in the Hippocampal Replay section above. Effectively, snippets close to a reward have more chance to be replayed and thus to be consolidated into a trajectory.

Panel A in Fig 6 illustrates the distribution of snippets selected from the two sequences, ABC in pink and ABD in blue. At the crucial point of choice at location B, the distribution of snippets for sequence ABC largely outnumbers those for sequence ABD. This is due to the propagation of rewards respectively from points C and D. Per design, rewards propagated from a more proximal location will have a greater influence on snippet generation. Panel C shows the 2D histogram of autonomously generated sequences when the model is primed with the initial sequence prefix starting at point A. We observe a complete preference for the shorter sequence ABC illustrated in panel E.

The snippet generation model described above takes into account the location of rewards, and the magnitude of rewards. Panel B illustrates the distribution of snippets allocated to paths ABC and ABD when a 10x stronger reward is presented at location D. This strong reward dominates the snippet generation and produces a distribution that strongly favors the trajectory towards location D, despite its farther distance. Panel F illustrates the error measures for model reconstruction of the two sequences and confirms this observation. This suggests an interesting interaction between distance and reward magnitude. For both conditions, distances to the expected sequence have been measured for every trajectory generated (10 000 for ABC and 10 000 for ABD). Then a Kruskall Wallis test confirms (p-value ~ = 0) for both cases that trajectories generated autonomously are significantly more accurate for the expected trajectories (i.e. ABC when rewards are equal and ABD when reward at D is x10).

### Novel efficient sequence creation

Based on the previously demonstrated dynamic properties, we determined that when rewards of equal magnitudes are used, the model would favor shorter trajectories between rewards. We now test the model’s ability to exploit this capability, in order to generate a novel and efficient trajectory from trajectories that contain sub-paths of the efficient trajectory. That is, we determine whether the model can assemble the efficient sub-sequences together, and reject the longer inefficient sub-sequences in order to generate a globally efficient trajectory. Fig 1 (Panel A) illustrates the desired trajectory that should be created without direct experience, after experience with the three trajectories in panels B-D that each contain part of the optimal trajectory (red), which will be used to train the model.

**Fig 7:**
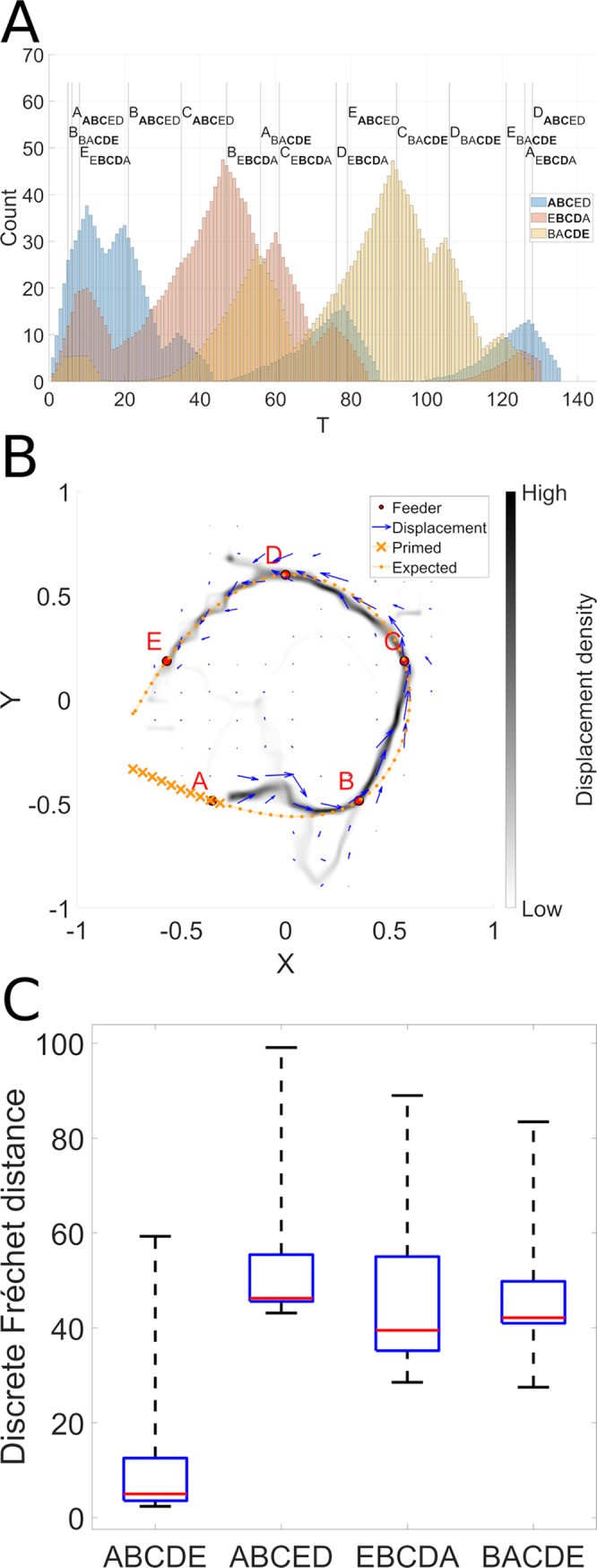
Efficient sequence synthesis. A. Distribution of snippets drawn from the sequences illustrated in Fig 1B, 1C and 1D. Globally we observe snippet selection favors snippets from the beginning of sequence ABCED (blue), the middle of EBCDA (yellow), and the end of sequence BACDE (pink), which corresponds exactly to the efficient sub-sequences (ABC, BCD, and CDE) of these three sequences. This distribution of snippets is used to train the model. The results of the training are illustrated in panel B. Here we see a 2D histogram of sequences generated by the model in the ABCDE recombination experiment. The 2D trajectory histogram is generated by superposing the trajectories generated when 10 batches of 100 reservoirs each were trained and each model instance was evaluated 10 times with noise. Panel C displays the Frechet distance between the autonomously generated sequence and the four reference sequences. Kruskal-Wallis comparison confirms that the trajectories generated autonomously are significantly more similar to the target sequence ABCDE than to the experienced non-efficient sequences (p < 0.0001).

The reward-biased replay is based on the following trajectories: (1) ABCED that contains the ABC part of the ABCDE target sequence, (2) EBCDA that contains the BCD part of the ABCDE target sequence, and (3) BACDE that contains the CDE part of the ABCDE target sequence. Fig 7A illustrates how the hippocampal replay model generates distributions of snippets that significantly favor the representation of the efficient sub-sequences of each of the three training sequences. This is revealed as the three successive peaks of snippet distributions on the time histogram for the blue (ABCED) sequence, favoring its initial part ABC, the yellow (EBCDA) sequence, favoring its middle part BCD, and the pink (BACDE) sequence, favoring its final part CDE. When observing each of the three color-coded snippet distributions corresponding to each of the three sequences we see that each sequence is favored (with high replay density) precisely where it is most efficient. Thus, based on this distribution of snippets that is biased towards the efficient sub-sequences, the reservoir should be able to extract the efficient sequence.

Reservoir learning is illustrated in Fig 7B, which displays the autonomously generated sequences for 1000 instances of the model executed 10 times each. Training is based on 1000 snippets of length 10 selected from the distribution illustrated in Fig 7A. The spatial histogram reveals that the model is able to extract and concatenate the efficient sub-sequences to create the optimal path, though it was never seen in its entirety in the input. Panel C illustrate the significant differences in performance between the favored efficient sequence vs. the three that contain non-efficient sub-sequences. A Kruskal-Wallis test confirms these significant differences in reconstruction error for the efficient vs non-efficient sequences (maximum p = 5.9605e-08). These robust results demonstrate that our hypothesis for efficient sequence discovery based on reward-modulated replay is validated.

### Reverse replay

In [1], hippocampus replay during SWR is characterized by the activation order of the place-cells which occurs in backward and forward direction. We hypothesize that reverse replay allows the rat to explore a trajectory in one direction but consolidate it in both directions. This means that an actual trajectory, and its unexplored reverse version, can equally contribute to new behavior. Thus fewer actual trajectories are required for gathering information for solving the TSP problem. A systematic treatment of this effect on learning can be seen in S1 Text Analysis of different degrees of reverse replay.

We now investigate how reverse replay can be exploited in a recombination task where some sequences are experienced in the forward direction, and others in the reverse direction, with respect to the order of the sequence to be generated. We use the same setup as described above for novel sequence generation, but we invert the direction of sequence EBCDA in the training set. Without EBCDA, the model is not exposed to sub trajectories linking feeders B to C and C to D and the recombination cannot occur. We then introduce a partial reverse replay, which allows snippets to be played in forward and reverse order. This allows the reservoir to access segments BC and CD (even though they are not present in the forward version of the experienced trajectory.

**Fig 8:**
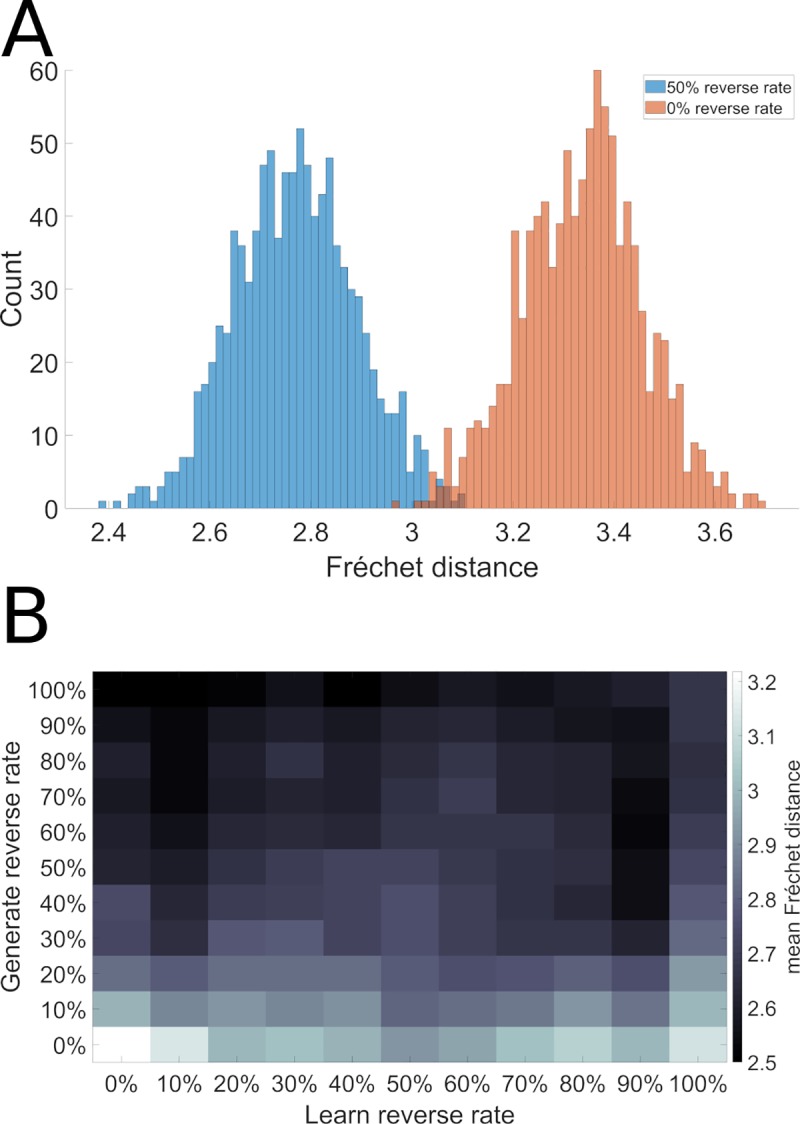
Reverse replay facilitates efficient sequence discovery. Using the same sequences illustrated in Fig 1, we reversed the direction of sequence EBCDA, and then tested the model’s ability to synthesize the ABCDE sequence from ABCED ACDBE and BACDE. A. Error reduction with reverse replay. B. Effects of reverse replay in generation and learning.

Fig 8 illustrates the histogram of sequence performance for 10000 runs of the model (1000 models run 10 times each) on this novel sequence generation task with and without 50% reverse replay. We observe a significant shift towards reduced errors (i.e. towards the left) in the presence of reverse replay.

We then examine a more realistic situation based on the observation of spontaneous creation of “shortcuts” described in [47]. The model is exposed to a random replay of snippets extracted from two trajectories having different direction (clockwise CW and counter clockwise CCW). The system thus experiences different parts of the maze in different directions. We examine whether the use of reverse replay can allow the system to generate novel shortcuts.

**Fig 9:**
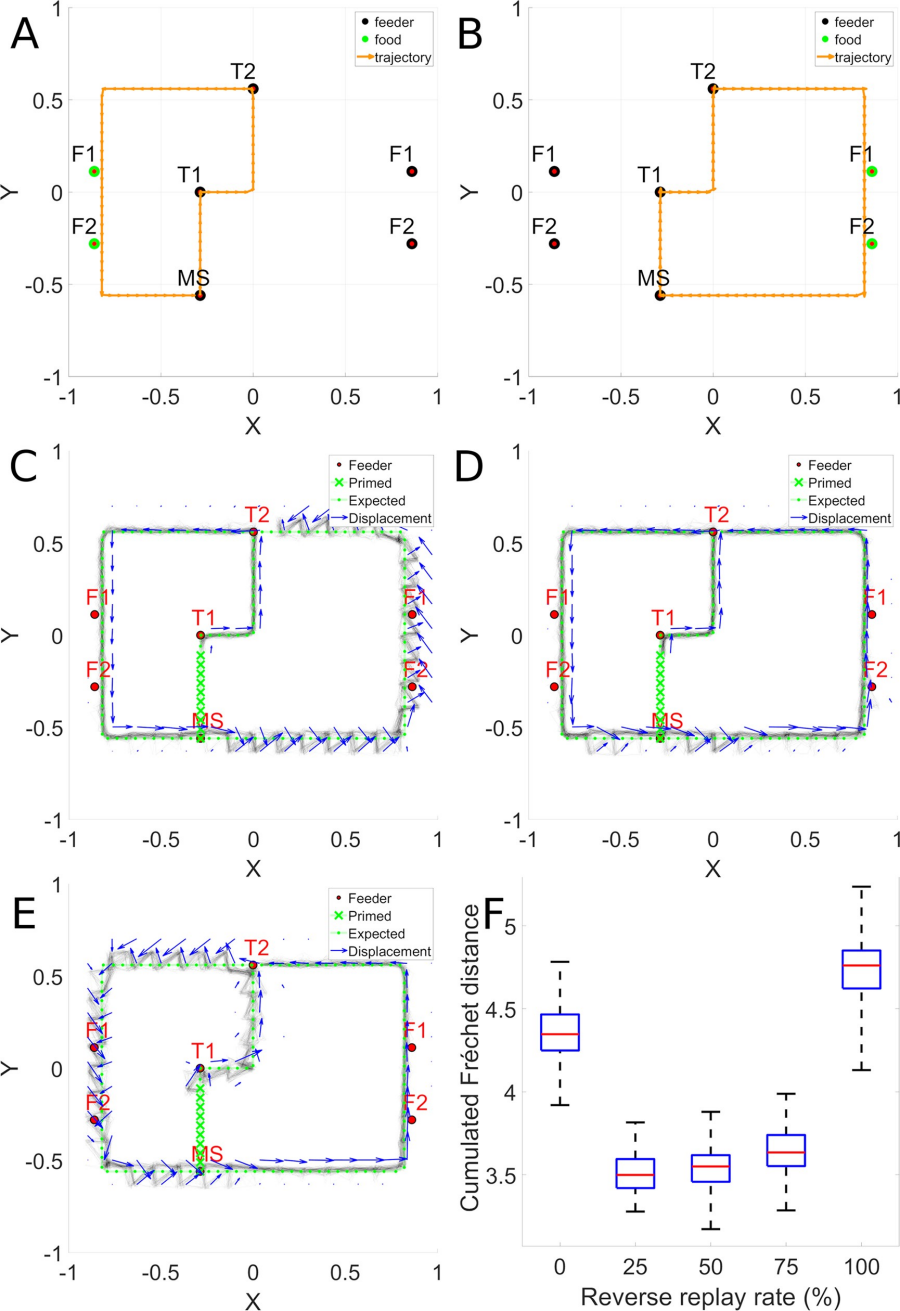
Reverse replay allows novel shortcut path generation. Panels A and B illustrate the trajectories for left and right trajectories, based on Gupta et al. After training on these two trajectories, we test the ability to generate a shortcut that makes the complete outer loop in one direction. Panel C–without reverse replay, significant spatial errors are revealed when the system attempts to complete the counter-clockwise loop on the right side of the maze. Panel D illustrates the beneficial effects of reverse replay during trajectory learning. Panel E illustrates the effect of a model training with 100% reverse replay. It is similar to using 0% reverse replay but the effect is observed on the left lap trajectory part. Panel F–when reverse replay is introduced, this error is attenuated.

The left and right trajectories used for training are illustrated in Fig 9A and 9B. In A, the system starts at MS, head up and to the left at T2 (counter clockwise) and terminates back at MS. In B, up and to the right (clockwise) again terminating at MS. Possible shortcuts can take place at the end of a trajectory at MS as the system continues on to complete the whole outer circuit rather than stopping at MS. We can also test for shortcuts that traverse the top part of the maze by starting at MS and heading left or right and following the outer circuit in the CW or CCW direction, thus yielding 4 possible shortcuts. The model is trained with snippets from the sequences in A and B using different random replay rates, and evaluated in non-autonomous mode with sequences representing the 4 possible types of shortcut. Fig 9C shows with no reverse replay, when attempting the CCW path, there is low error until the system enters the zone that has only been learned in the CW direction. There, the system displays clear deviations from the desired path. In the non-autonomous evaluation mode used in this experiment, after each response, the system is provided with the desired next location, which in this case creates a zigzag effect, corresponding to the spatial error. In panel D, with 50% reverse replay, this error is reduced and the system can perform the shortcut without having experienced the right hand part in the correct direction. Thus, in the right hand part of the maze, it is as if the system had experienced this already in the CCW direction, though in reality this has never occurred, but is simulated by the reverse replay. This illustrates the utility of mixed forward and reverse replay. Panel E illustrates the difficulty when 100% reverse replay is used. Fig 9F illustrates the reconstruction errors for a shortcut path as a function of degree of reverse. The trajectory is evaluated in non-autonomous mode and the position of the agent necessarily follows the target trajectory. In this case, the expected trajectory describes a CCW path. Results are not significantly different with a replay rate 25% and 75% (p = 0.02), where the best performance is observed, and all the other conditions are significantly different (p ≤ 1.1921e-07). This phenomenon was obtained for the 4 possible shortcuts.

### Effects of consolidation and reverse replay

The model demonstrates the ability to accumulate and consolidate paths over multiple trials, and to exploit reverse replay. Here we examine these effects on the more extensive and variable dataset extracted from rat behavior [13]. We show the positive effects of replay on trajectories from rats trying to optimize spatial navigation in the TSP task. In the prototypical TSP behavior, in a given configuration of baited wells, on successive trials the rat traverses different efficient sub-sequences of the overall efficient sequence, and then finally puts it all together and generates the efficient sequence. This suggests that as partial data about the efficient sequence are successively accumulated, the system performance will successively improve. To explore this, the model is trained on navigation trajectories that were generated by rats in the TSP task. We selected data from configurations where the rats found the optimal path after first traversing sub-sequences of that path in previous trials. Interestingly, these data contain examples where the previous informative trials include traversal of part of the optimal sequence in either the forward or reverse directions, and sometimes both (see S Rat navigation data). We trained the model with random replay of combinations of informative trials where informative trials are successively added, in order to evaluate the ability of the model to successively accumulate information. For each combination of informative trials, the random replay is evaluated with 0%, 25%, 50%, 75% and 100% of reverse replay rate in order to assess the joint effect of random replay and combination of informative trials. The model is then evaluated in non-autonomous mode with the target sequences that consist in a set of trajectories linking the baited feeders in the correct order. An idealized sequence is added to the target sequence set because trajectories generated by the rat might contain edges that do not relate the shortest distance between two vertices. Agent’s moves are restricted to a circle having a 10 cm radius.

**Fig 10:**
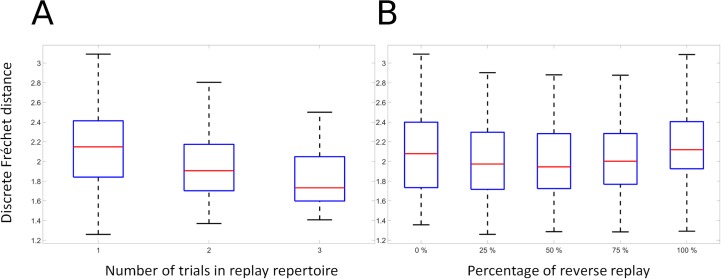
Consolidation and reverse replay applied to behavioral data. Measured variable is Frechet distance between generated and desired sequence. Data from the rat TSP configurations are used for training and testing the model. A. Effects of consolidation: as successive trials are added to the replay repertoire; the trajectory reconstruction error is significantly reduced. B. Effects of reverse replay: as reverse replay is introduced in snippet formation for training the PFC model, reconstruction error is significantly reduced.

Fig 10 illustrates the combined effects of successive integration of experience and its contribution to reducing error, and of the presence of different mixtures of forward and reverse replay. The ANOVA revealed that there is a significant effect for combination (F(2, 585) = 32.84, p < 0.01), as performance increases with exposure to more previous experience (Panel A). There is also a significant effect for reverse replay rate (F(4, 585) = 3.71, p ≤ 0.01), illustrated in Panel B. There was no significant interaction between consolidation and replay direction (F(8, 585) = 0.03, p = 1). This indicates that when trained on trajectories produced by behaving rats, the model displays the expected behavior of improving with more experience, and of benefitting from a mixture of forward and reverse replay.

## Discussion

We tested the hypothesis that hippocampus replay observed during sharp wave ripple events in the awake animal can play a role in learning by exposing the prefrontal cortex between successive trials to short sub-sequences of place-cell activation patterns. This replay can potentially play a crucial role in learning, essentially by generating synthetic data (based on experience) for training the system. The behavior of interest is a form of spatial navigation trajectory optimization in a task, mimicking the well-known traveling salesperson problem. It is a NP-Hard (non-polynomial) problem and finding an exact solution would require significant time and computing resources. Nevertheless, it has been observed that a rat was able to quickly find good solutions of simplified versions of this problem [13, 48]. The idea of exploiting replay in navigation sequence learning has been demonstrated to have a positive influence on learning [17], and here we go beyond this by further exploiting reward structure in the replay.

In the behavior of interest, rats are observed to converge quickly to a near-optimal path linking 5 baited food wells in a 151cm radius open arena. During their successive approximation to the optimal path, the rats often traversed segments of the optimal trajectory, as well as non-optimal segments. Observing this behavior, we conjectured the existence of neural mechanisms that would allow the optimal segments to be reinforced and the non-optical segments to be rejected, thus leading to the production of the overall near-optimal trajectory. We propose that the overall mechanism can be decomposed into two distinct neural systems. The first is a replay mechanism that favors the representation of snippets that occurred on these optimal segments, and that in contrast will give reduced representation to snippets that correspond to non-optimal trajectory segments. Here we demonstrate a simple but powerful method based on spatial reward propagation that implements this mechanism. Interestingly, this characterization of replay is broadly consistent with the effects of reward on replay observed in behaving animals [9].

The second neural system required to achieve this integrative performance is a sequence learning system that can integrate multiple sub-sequences (i.e. snippets) into a consolidated representation, taking into consideration the probability distributions of replay so as to favor more frequently replayed snippets. Here we considered a well-characterized model of sequence learning based on recurrent connections in prefrontal cortex that is perfectly suited to meet the sequence learning requirements.

### Replay mechanism

Replay is modeled using a procedure that randomly selects a subset of place-cells coding part of a sequence, and outputs this snippet while taking into account the proximity of this snippet to a future reward. Each time a reward is encountered, it is taken into consideration in generating the snippet, and reward value is propagated backwards along the sequence, thus implementing a form of spatio-temporal credit assignment. This can be viewed in the Figs 2, 6 and 7 illustrating the snippet probability densities. The replay mechanism also implements a second feature observed in animal data, which is a tendency to replay snippets in reverse order. These two features of the replay model correspond to what is observed in the rat neurophysiology, and they also make fundamental contributions to the model’s ability to converge on an efficient navigation path. This extends previous demonstrations of the value of replay to include reward-modulated optimization [17].

### Reservoir network

Reservoir computing exploits the spatio-temporal dynamics of recurrently connected neurons that are sensitive to the spatiotemporal structure of input sequences [20, 27, 28]. The frontal cortex has been demonstrated to operate on these reservoir properties [19]. Here we demonstrated how a reservoir model of PFC meets two requirements for sequence learning: First, it can concatenate randomly replayed sub-sequences (snippets) in order to generate the complete original sequence. Second, it is sensitive to the statistics of replay, and thus can learn to ignore rare snippets (which correspond to snippets on inefficient sub-sequences, far from rewards) thus learning to optimize.

### Effects of reward and reinforcement learning

The instantaneous reward information acquired during a past experience is used for recursively updating the snippet replay likelihood in the hippocampus model. This creates a reward gradient and allows the optimal sequence to be assembled by the prefrontal cortex and striatum model. This is a novel combination of prioritized replay and reservoir computing in the context of reinforcement learning. The reward gradient is propagated along the spatial trajectory, and used to create a bias in the probability of replay. This biased replay is then provided as input to the reservoir PFC model. This is complementary to [49] who used replay to train a Dyna-Q reinforcement learning model. Both models benefit from replay, and can adapt to changes in reward contingencies. In our system, when the distant feeder is given a higher reward, this large reward produces a shift in replay probabilities (illustrated in Fig 6B), and the model learns this new distribution and favors the longer path to target D (illustrated in Fig 6D). The distinction is that we modulate the replay by reward probability, thus biasing the input to the sequence learning model towards the optimal solution. A secondary effect of rewards could be observed when rewards are sufficiently close for allowing a mutual contribution to the snippet replay likelihood surrounding the locations associated with reward delivery. Thus, we predict that a cluster of reward sites will have the effect of propagating the reward information farther than a single reward.

While we were principally motivated to study reward-prioritized replay combined with reservoir sequence learning in the TSP task, one can ask if the model generalizes to other tasks, particularly those that directly involve manipulation of reward. We thus observed that by changing the reward magnitudes, the system adapts and chooses a longer trajectory that leads to a larger reward. However, in more difficult problems that include the discovery of a long route to a single reward, the model could participate in the consolidation of partial solutions as illustrated in the current research, but would not be able to solve such problems autonomously.

### Effects of reverse replay

The reverse replay mechanism has a dual effect. First, it provides the mechanism for the backwards propagation of reward along a trajectory. Based on this reward propagation, place-cell activation sequences leading to a nearby reward are represented more frequently and earlier than other less efficient sub-paths, which are thus rejected. This results in a form of spatio-temporal credit assignment that allows to take advantage of the reservoir network ability to combine multiple snippets into a whole sequence. We showed that it is possible to consolidate multiple sequences featuring parts of the same underlying optimal sequence into one efficient sequence and to generate it autonomously. Second, when the snippet replay likelihood is learned, a non-zero reverse replay rate allows the prefrontal cortex to be exposed to sequences of place-cell activations in both forward and reverse direction. This results in sequence learning in both directions while having experienced a place-cell activation sequence in one direction only. These results can be tested experimentally by recording place cells activities in SWR during the task.

### Predictions

During the intertrial period, the model predicts a co-occurrence of reverse replay from remote rewarded sites backwards to propagate the reward, and forward replay from remote locations towards rewarded sites to generate snippets from the optimal sub-sequences so as to generate the optimal path. Importantly, it also predicts that there will be a low probability of replay for subsequences that were on non-optimal trajectories. Future research should test these predictions.

A model of replay should predict which experiences should be replayed at each time to enable the most rewarding future decisions. Mattar and Daw [50] developed an elegant model of replay based on utility, characterized by a gain term that prioritizes states behind the agent when an unexpected outcome is encountered and a need term that prioritizes states ahead of the agent that are imminently relevant. This model predicts predominantly forward sequences prior to a run, and reverse sequences after a run. It accounts for a wide variety of behavioral and neurophysiological data, often in protocols where replay is observed during a run. We address a problem where the system is in a neutral area between trials in the TSP task. Thus, the current position of the animal is of low relevance. In this context, our model replays snippets that lie on the shortest route through the five baited paths. It would be interesting to observe how the Mattar and Daw model would respond during intertrial intervals in resolving the TSP problem.

### Conclusions and limitations

The model we studied here is able to mimic the rat’s ability to find good approximations to the traveling salesperson problem by taking advantage of recent rewarding experiences for updating a trajectory generative model using hippocampus awake replay. We showed that reverse replay allows the agent to reduce the TSP task complexity by considering an undirected graph where feeders are vertices and trajectories are the edges instead of a directed graph. In this case, autonomous sequence generation is no longer possible but the information available in each prediction of the prefrontal cortex contains the expected locations. This allows the building of a navigation policy taking into account the salient actions suggested by the prefrontal cortex predictions, which are learned from hippocampus replay.

## Supporting information

S1 Text This supporting information provides details on the rat navigation data, and the functioning of the model, including high dimensional processing in the reservoir, the spatial filter implementation and parameters, the sensor and transition model components of the simulation system, the Frechet distance parameters, basic sequence learning and parameter search, the effects of sequence complexity on consolidation, analysis of different degrees of reverse replay. (PDF)

S1 Fig Rat trajectory and its idealized representation. (TIF)

S2 Fig Internal representations in the reservoir. (TIF)

S3 Fig Spatial filter characterization. (TIF)

S4 Fig Fréchet distance visualization. (TIF)

S5 Fig Spatial resolution vs. reservoir neuron leak rate. (TIF)

S6 Fig Effects of snippet size on sequence learning for sequences of varying difficulty. (TIF)

S7 Fig Effects of reverse replay and initial sequence direction. (TIF)

S1 Table Classification of rat behavioral configurations by direction in which executed trajectories relate to desired trajectory. (DOCX)

S2 Table Summary of the 10 parameter sets optimized by the parallel simulated annealing algorithm. (DOCX)

S3 Table Performance (revealed by Fréchet distance to desired trajectory) as a function of snippet size. (DOCX)

S1 Data Cazin-model-data–Model and data to run selected experiments. (ZIP)
