# Supplementary material for: Reservoir computing model of prefrontal cortex creates novel combinations of previous navigation sequences from hippocampal place-cell replay with spatial reward propagation
Source: PLoS Comput Biol. 2019 Jul 15;15(7):e1006624. doi: 10.1371/journal.pcbi.1006624 (PMC6668845; doi:10.1371/journal.pcbi.1006624)
Supplement: S1 Text — (PDF) [file pcbi.1006624.s001.pdf]

Supplementary Material S1 for Cazin et al.

Rat navigation data

Navigation trajectories are sampled from 100 different configurations of baited feeders that were explored by different rats over 10 trials each. Before each trial, the rats were positioned at the same starting point and the trajectory of the animal is recorded by an overhead camera at about 20-30 frames per seconds. Figure 1 panel A depicts the trajectory performed by a rat during trial 4 for configuration 84 (17,10,3,14,15).

INSERT Figure 1 HERE

Video tracking sometimes contain noisy or missing samples and thus we interpolate data with splines. Trajectories are first condensed by eliminating contiguous samples sharing the same coordinates (i.e. static parts of the trajectories are pruned). Then the remaining points of the trajectories are resampled evenly in order to match the target spatial resolution of 20 $points * m^{-1}$, corresponding to 5cm between each position of the trajectory. This can be visualized in Figure 2B.

In the acquired dataset of 100 configurations of rat behavior, 64 configurations resulted in the rat finding the optimal paths. In 2/3 (42/64) of these configurations the animal found the optimal trajectory without having previously experienced the component subsequences. In one third (22/64) of the optimal configurations the animal traversed parts of the efficient path in early trials before finally displaying the efficient trajectory. These data are thus suitable for testing our hypothesis, and are used in our experiments. For these data we created an idealized trajectory that link optimally the baited feeders as illustrated in Figure 1, panel B. This is required for establishing a performance criterion to assess the model.

By analyzing the trajectories, it is possible to split the dataset in 9 different groups characterized by two criteria: The number of informative trials required to observe all the edges contained in the target trajectory, and the direction of the edges contained in the trials taken into account

INSERT Table 1 HERE

In Table 1 each column contains configurations in which the rat performed trajectories containing edges of the target trajectory in a particular direction, and in which the rat converged on a near-optimal trajectory at least once. Each configuration consists of 8 to 13 trials where each trial might contain a trajectory having a shared edge with the target trajectory. Such a trial is called an informative trial. Therefore, a given configuration can be classified into different clusters. For example, configuration 97 has three trials that contain the edges of the optimal sequence in the forward direction and three trials containing the edges of the optimal sequence in the forward and backward directions. Configurations 80 and 58 contain three trials in which trajectories have edges in the backward direction only.

High dimensional processing in the reservoir

Reservoir states carry information about its present and past activation values within a limited time span. One single reservoir neuron does not contain the whole information but its activation fluctuations carry partial information about the serial and temporal structure of the stimulus sequence. This property is related to mixed selectivity (Rigotti, Barak et al. 2013).

The concatenation of b reservoir states from time t_n to time t_{n+b} is noted $X_{res}(t_n \rightarrow t_{n+b})$. If we attempt to represent the mean firing rate activation pattern of neurons within this time range, we can observe that the dynamics are rich but it is difficult to read useful information from a raster: Figure 2 shows in panel B the raster of reservoir neurons when exposed to the ABCDE place-cell activation sequence depicted in Panel A.

INSERT Figure 2 HERE

Instead, we propose to compute the Singular Value Decomposition (SVD) of the matrix $X_{res}(t_n \rightarrow t_{n+b})$. $X_{res}(t_n \rightarrow t_{n+b})$ is viewed as a matrix containing b observations of N variables

(number of neurons in reservoir). The SVD factorizes $X_{res}(t_n \rightarrow t_{n+b})$ into three matrices named U, Σ, V such that:

$$X_{res}(t_n \rightarrow t_{n+b}) = U * \Sigma * V^*$$

(1)

We will call the matrix $U * \Sigma$ the "equivalent reservoir". Figure 2.C shows a representation of the neural network model including the equivalent reservoir.

Matrices U and V^*might be viewed intuitively as rotation matrices and Σ as a scaling matrix. Since U and $V^*$are orthonormal basis, we can see $U * \Sigma = X_{res}(t_n \rightarrow t_{n+b}) * V$ as a raster of equivalent neurons. Each equivalent neuron is a neuron whose activation value is a linear combination of reservoir neurons. The connectivity matrix that projects X_{res} reservoir neurons into equivalent reservoir neurons X_{eq} is matrix V.

$$X_{eq}(t_n \rightarrow t_{n+b}) = X_{res}(t_n \rightarrow t_{n+b}) * V$$

(2)

In Figure 2.D, we observe the matrix $X_{eq}(t_n \rightarrow t_{n+b})$ as a raster of equivalent neurons. Most of the information carried by reservoir neurons can be described with a significantly lower number of dimensions.

This will allow us to interpret reservoir neurons activation patterns as a simple trajectory in a two or three -dimensional space as depicted in Figure 2.E

The activation values of the first three neurons of the equivalent reservoir over time are represented as a three-dimensional trajectory. Time steps of the trajectory that match with a feeder position is represented with the feeder associated label (A, B, C, D or E).

Moreover, the W'_{ro} synaptic weights that connect the equivalent reservoir to the readout layer are exactly the correlation coefficient between equivalent reservoir and readout neurons.

$$W_{ro} = V * corr(X_{des}, X_{eq})$$

Intuitively, the readout layer selects the linear combinations of neurons correlated with the expected activation pattern.

Spatial Filter implementation and parameters

The spatial filter is used to transform potentially noisy place cell codes in the reservoir output into their corresponding location, and then recoding this in a clean and well-formed place cell code. By using Bayes rule, we can write:

$$P(s(t_{n+1})|\eta(t_n), s(t_{n-1})) = \frac{P(\eta(t_n)|s(t_{n+1}))P(s(t_{n+1})|s(t_n))}{P(\eta(t_{n+1})|s(t_n))}$$

(3)

Where:

- $P(\eta(t_n)|s(t_{n+1}))$is called the sensor model and represents the probability to fire the pattern η at time t_n, given the point s at time t_{n+1}
- $P(s(t_{n+1})|s(t_n))$ is called the transition model and represents the probability for the agent to be at location $s(t_{n+1})$ given the current location $s(t_n)$
- $P(\eta(t_n)|s(t_n))$is a normalization factor that does not depend on $s(t_{n+1})$/

The sensor and transition model need to be defined in order to evaluate the most probable point given the predicted place-cell activation vector and the current position.

Sensor model

If we consider the place-cell activations $\eta = (\eta_k)_{k\in 1\ldots K}$ at time t as a conditionally independent consequence and the site $s = (x, y)$ as the cause that generated η, the sensor model can be written as:

$$P(\eta|s) = \prod_{k=1}^{K} P(\eta_k|s)$$

(4)

We propose to estimate the posterior probability $P(\eta|s)$ by using a similarity function based on a one-dimensional Gaussian function for mapping any difference between mean firing rates $f_k(s)$ and η_k into a real number between 0 and 1. The Gaussian based function is described by the equation:

$$g(x, x'; \sigma) = e^{-\frac{(x-x')^2}{2\sigma^2}}$$

(5)

Where:

- σ is the standard deviation of the Gaussian function

Thus, when a mean firing rate $f_k(s)$ is close to η_k, the corresponding response $g(f_k(s), \eta_k; \sigma)$ is close to 1. A very different mean firing rate will be related with a response close to zero. Parameter σ controls the selectivity of the kernel. Small σ values will define a narrow Gaussian curve and result in the rejection of points separated from a relatively close distance from each other. We define the posterior probability as:

$$P(\eta_k|s) = \frac{g(f_k(s), \eta_k; \sigma)}{\sum_{s' \in S} g(f_k(s'), \eta_k; \sigma)}$$

(6)

Then it is possible to rewrite equation (4) as:

$$P(\eta_k|s) = \prod_{k=1}^{K} \frac{e^{-\frac{(f_k(s)-\eta_k)^2}{2\sigma^2}}}{\sum_{s' \in S} e^{-\frac{(f_k(s')-\eta_k)^2}{2\sigma^2}}}$$

(7)

Since we are using a product of exponential functions, we can simplify equation (4) :

$$P(\eta|s) = e^{-\frac{\|f(s)-\eta\|^2}{2\sigma^2}} \Gamma(\eta)$$

(8)

Where:

- $\Gamma(\eta) = \prod_{k=1}^{K} \frac{1}{\sum_{s' \in S} e^{-\frac{(f_k(s')-\eta_k)^2}{2\sigma^2}}}$ is a normalization function that only depends on η

$\Gamma(\eta)$ might be simplified by considering that $\sum_{s' \in S} e^{-\frac{(f_k(s')-\eta_k)^2}{2\sigma^2}}$ is an approximation of the one dimensional Gaussian integral. The Gaussian integral in K dimensions is given by:

$$\int_{\mathbb{R}^K} e^{-\alpha\|x\|^2}\, dx = \left(\frac{\pi}{\alpha}\right)^{\frac{K}{2}}$$

(9)

Where:

- $x = f_k(s) - \eta_k$
- $\alpha = \frac{1}{2\sigma^2}$

Thus, the one-dimensional Gaussian integral approximation evaluates to a constant $\sum_{s'\in S} e^{-\frac{\left(f_k(s')-\eta_k\right)^2}{2\sigma^2}} \approx (2\pi\sigma^2)^{\frac{1}{2}}$ and we have:

$$\Gamma \approx (2\pi\sigma^2)^{-\frac{K}{2}}$$

(10)

In equation (8), $\Gamma(\eta)$ could be also defined as:

$$\Gamma(\eta) = \frac{1}{\sum_{s'\in S} e^{-\frac{\|f(s')-\eta\|^2}{2\sigma^2}}}$$

(11)

According to equation (9), the K-dimensional Gaussian integral approximation evaluates to $\sum_{s'\in S} e^{-\frac{\|f(s')-\eta\|^2}{2\sigma^2}} = (2\pi\sigma^2)^{\frac{K}{2}}$ and we still have $\Gamma \approx (2\pi\sigma^2)^{-\frac{K}{2}}$. It is also possible to evaluate $\Gamma(\eta)$ with taylor series expansions but the major drawback of this solution would be its sensitivity to noise because of the use of high order polynomials. Since we are only going to evaluate all the points of the S 2D space and find the maximum of equation (8), the normalization constant Γ could be omitted. The computation equation (8) is based on a similarity function G which measures the similarity between place-cell activation patterns:

$$G(x, x') = e^{-\frac{\|x-x'\|^2}{2\sigma^2}}$$

(12)

It is a K dimensional Gaussian kernel similar to those used in support vector machines **(Cortes and Vapnik 1995)**. Intuitively, it is a dot product in feature space. In Figure 3, panel A shows an example of the values of equation (12) computed for $\sigma = 10$ and a finite element map of 1000x1000 elements. The most probable locations emerge as the most salient areas.

INSERT Figure 3 HERE

Note that the same selection principle could be extended to any place-cell activation spatial response. A two-dimensional grid description of a place-cell activation function built on observations gathered during a behavioral experiment with a rat is a good example.

Transition model

The agent can move only on its adjacent locations. This is a constraint of physical continuity that restricts the areas of the map that must be evaluated for selecting the next location $s(t_{n+1})$. This is modeled by a circle of radius R centered on the agent's current position $s(t_n)$. The agent is allowed to move within a circular sector defined by Radius R and angle α defined by:

$$V = \left\{ s(t_n) \in S \,\middle|\, ||\vec{c}(t_n)||^2 < R^2 \; and \; \frac{< \vec{c}(t_n), \vec{d}(t_{n-1}) >}{||\vec{c}(t_n)|| ||\vec{d}(t_{n-1})||} < \cos(\frac{\alpha}{2}) \right\}$$

(13)

Where:

- $\vec{c}(t_n)$= $s(t_n)$- $s(t_{n-1})$ is a vector that represent the 'heading' of the agent, based on its current position $s(t_n)$ and previous position $s(t_{n-1})$
- $\vec{d}(t_n)$= $s(t_{n+1})$- $s(t_n)$ is a vector that represent the future heading of the agent, based on the future position $s(t_{n+1})$ being evaluated and its current position $s(t_n)$

If no direction is available (i.e. the agent is no longer moving) or if α is set to zero, only the $||\vec{c}(t_n)||^2 < R^2$condition is evaluated. R is chosen in order to enclose the maximum range of motion of the agent. Restricting the search space for the most probable point by using an appropriate radius R bounds and significantly reduces the time complexity of the implementation. We then define the transition model as:

$$P(s(t_{n+1})|s(t_n)) = \begin{cases} \frac{1}{|V|} \; if \; s(t_{n+1}) \in V \\ 0 \; otherwise \end{cases}$$

(14)

Where $|V|$ represents the area of the V set

Once again, we can omit the normalization constant because of the maximization problem we are trying to solve and the term $\frac{1}{|V|}$in (14) can be replaced by 1

A unified formulation

In (Zhang, Ginzburg et al. 1998) several reconstructions methods are evaluated and they can be viewed as the maximization of a function $\psi(s,\eta)$ defined by the following general equation for K place-cells:

$$\psi(s,\eta) = \sum_{k=1}^{K} \phi_k(s) * \eta_k + A(s)$$

(15)

Where η_k is the mean firing rate of place-cell k, $\phi_k(s)$is a basis function associated with place-cell k and $A(s)$ is a bias term that is independent of place-cells and reminiscent of a regularization term.

The sum over the K place-cells is in fact a dot product and equation (15) can be rewritten as:

$$\boldsymbol{\psi}(\mathbf{s},\boldsymbol{\eta}) = \mathbf{K}(\boldsymbol{\phi}(\mathbf{s}),\boldsymbol{\eta}) + \mathbf{A}(\mathbf{s})$$

(16)

Where $K(x,x')$ is a function that evaluates the similarity between x and x'

In (Zhang, Ginzburg et al. 1998), the similarity function $\mathrm{K}(x,x')$ is the usual dot product $< x,x' >$.

Reconstructing a two-dimensional location s^*based on place-cell activation pattern is equivalent to maximizing the similarity function $K(\phi(s),\eta(t))$ under the constraint represented by $A(s)$. The reconstruction problem is stated as:

$$s^*(t) = argmax_{s\in S}(\psi(s,\eta))$$

(17)

We propose to formulate our reconstruction kernel based/Bayesian method by using this unified formulation and identifying terms in equation **(16)** where $\phi(s) \equiv f(s)$ is the firing rate map (i.e. the spatial response of the place-cells), $K(x, x') \equiv G(x, x')$ is the Gaussian kernel defined in equation (12), $A(s) \equiv u$ is the noise function, $S \equiv V$ is the set if points lying in a circle sector centered on the agent as defined in equation (13)

Parameterization of the Spatial Filter

The spatial filter is required for evaluating the model and it is necessary to evaluate its accuracy. We use a finite element method for simplicity and speed. The two-dimensional space that encloses the arena ($4m^2$) is thus discretized into square tiles of equal size. Depending on the spatial resolution of this uniform grid, trajectories discretized through this grid will exhibit an aliasing effect if the grid size is too coarse. We estimated the absolute discretization error by measuring repeatedly the distance between a random point drawn uniformly in [-1,1] x [-1,1] and its discretized coordinates.

A grid of 1024 x 1024 provides an acceptable discretization error of less than 1.5 millimeter

Place-cell prediction might be noisy and it is useful to evaluate the performances of the spatial filter in noisy conditions. For each possible grid size, an important number of random points in [-1, 1] x [-1, 1] is drawn from a uniform distribution. Then for each point, a uniform noise is added to the mean firing rate patterns of place-cells activations that correspond to random points. The noise magnitude varies from 0 to 1 (equivalent to 100% mean firing rate) and produce different values of signal to noise ratio.

The reconstruction error is measured for different values of the σ parameter of the spatial filter (described in section Sensor model) and for different values of signal to noise ratio. Figure3, panel B shows the reconstruction mean square root error of different place-cell location decoding methods. We can observe that the linear model is quite poor at decoding an approximation of a hippocampus place-cell activation. Even with a 0.03 noise magnitude (SNR = 13.64dB, 3% error in place-cell pattern prediction) added to the mean firing rate of 256 place-cells, the linear decoder commits errors about 10 cm. Our kernel-based decoder provides reconstruction errors about the same order of magnitude as the discretization error when no noise is added. Reconstruction error is still under 1cm with a 10% noise

(SNR = -0.33dB). Panel C summarizes the performance of our decoder for different combinations of σ and noise magnitude.

It appears that $\sigma = 10$ is the best parameter regardless of the grid size. The spatial filter will now be configured with a 1024 x 1024 grid and $\sigma = 10$.

Fréchet distance parameters

Intuitively, the Fréchet distance is the maximum distance between matched points of curves A and B. Figure 4 illustrates the discrete Fréchet distance.

INSERT Figure 4 HERE

The closest points of curves A and B are matched by finding the sequence of steps along A and B (i.e. coupling) that must be followed to achieve the minimum coupling distance. The free space diagram depicted in panel A represents the propagation of the reachability information between two points of A and B.

A point in the coupling sequence associates two steps of sequence A and B whose Euclidian distance minimize the coupling distance.

In calculating the Fréchet distance, the $max(.,.)$ function is used in order to measure the coupling distance between A and B. A particular case happens when A and B are similar, excepted at unique step t. The distance between curves at this step will be reported as the Fréchet distance and we prefer to avoid masking interesting results by using a variant of the Fréchet distance. Instead of $max(.,.)$, we use the $sum(.,.)$ function (i.e. operator +). The implied coupling distance gives the minimum of the total distance of an order preserving correspondence between points of P and Q (Eiter and Mannila 1994).

Basic Sequence learning and parameter search

The model performance depends on several parameters. In (Lukosevicius 2012), a guideline for manual parameters tuning is described. Tuning parameters like input scaling requires experience and intuitive insight, and trial and error is suggested. Trial and error is also suggested for tuning the leak rate parameter and the spectral radius of the recurrent weights matrix should be tuned according to the mnesic

abilities required by the task. We prefer to rely on automated parameter selection and we use a clustered simulated annealing algorithm as described in (Ram, Sreenivas et al. 1996). A solution is thus a particular set of parameters for the model. The possible values of those parameters are:

- W_{in} scaling factor having values from 0 to 10 with 0.001 increments and noted in_scale
- W_{rec} scaling factor having values from 0 to 10 with 0.001 increments and noted rec_scale
- $leak\ rate$ having values from 0 to 1 with 0.001 increments and noted $leak\_rate$

In section Reservoir model of PFC for snippet consolidation, we defined $S(N;K) = K\frac{1}{\sqrt{N}}$ as the scaling factor of W_{rec} with N being the number of neurons in the reservoir because the spectral radius $\rho(W_{rec})$ is approximately the same for different values of N. In the parameter search, we are looking for the optimal value of rec_scale such that $S(N; rec\_scale) = rec\_scale\frac{1}{\sqrt{N}}$

This parameter set is evaluated on $B = 10$ different model instances, and Fréchet distance measurements are reduced into a one-dimensional cost function (described in equation 37) that is associated to the parameter set.

A population of C$= 10$ solutions is evaluated during $I = 1000$ iterations. The initial temperature is $T_0 = 1$ and the final temperature $T_I = 1 * 10^{-20}$. The cooling schedule is exponential multiplicative ($T_k = T_0 * \alpha^k$) and $\alpha = (\frac{T_I}{T_0})^{\frac{1}{I}}$. Every $K = 100$ iterations, the population is renewed with mutations of the best solution evaluated so far. The number of reservoirs neurons is set to $N = 1024$. The noise injected in the location probability map (see paragraph Embodied Simulation of Sensory-Motor Loop) is set to $m = 0$ in order to ensure that the next location selected during the sequence generation is the most probable location. Each model instance is trained with a slow learning rate of $\alpha = \frac{0.1}{N} = 9.7656e - 05$ and by exposing only 100 times to the same sequence.

As a preliminary experiment, we propose to search for a parameter set that allows the model to learn and generate a complex navigation sequence. A sequence is qualified as complex sequences because it contains loop, a feature that is particularly challenging for a sequence learning model. In the main text, Figure 4 panel A illustrates a complex trajectory featuring 2 loops. It corresponds to a navigation task

requiring good mnesic abilities and from a simulation point of view, sequences with long loops require a context, or state history, long enough to disambiguate repeating subsequences. The goal is to find a parameter set that allows learning and generating complex sequences even in degraded conditions. Table 2 summarizes the results of 10 parallel simulated annealing procedures.

INSERT Table 2 HERE

The best solution has been evaluated with a score of 1.1879078. It means that the sum of coupling distances of the generated sequences is low for every model instance evaluated. In the main text, Figure 4 panel B shows the trajectories generated by the 10000 instances of model setup with the best parameters. They correspond visually to the expected trajectory despite the fact that noise has been added for an easier visualization. We propose to estimate the position prediction error as an average coupling distance between generated and target Trajectory by computing for the 10000 model instances the discrete Fréchet distance score (sum of coupling distances) and to divide it by the number of samples in the target trajectory. It results in a set of averages of distances to the target trajectory. After exposing the model only 100 times to the target sequence, it is able to generate it with a prediction error of 1.16cm ± 2.4mm. A longer exposure of 1000 times to the target sequence lowers the prediction error to 1.13cm ± 2mm and. There is no significant gain at exposing the model 10 times longer to the training set.

The discretization error induced by the use of a finite element method for location reconstruction with our place-cell decoding Bayesian method (see Embodied Simulation if Sensory-Motor loop in main text) is 1.5mm. It is the lower boundary of the prediction error it is possible to reach. The reconstruction error of our Bayesian method with a noise free signal is 0.32mm ± 0.22mm

This demonstrates that the overall model with only 1024 reservoir neurons is suitable for trajectory learning and the best parameter set is $in\_scale = 6.379720$, $rec\_scale = 6.553708$, $leak\_rate = 0.494997$. The optimized $rec\_scale = 6.553708$ value results in a spectral radius $\rho(W_{rec}) \approx 3.8712$ and $in\_scale = 6.379720$ ensures a significant influence of the input on the reservoir dynamics. The inverse of the model time constant, $leak\_rate = 0.494997$, is adapted to the dynamics of the complex training sequence sampled at a spatial resolution of 20 $dots * m^{-1}$.

By keeping the same parameters and trajectory and then varying the $leak\_rate$ from 0 to 1 and the spatial resolution of the trajectory, it is possible to show that there exists for each spatial resolution a tight range of leak rates that allows good performance. Figure 5 illustrates this property of recurrent neural networks.

INSERT Figure 5 HERE

The score of each $(leak\ rate, spatial\ resolution)$ pair is depicted on a heat map with a logarithmic scale. The x-axis represents the spatial resolution in $dots * m^{-1}$ and the y axis the leak rate $leakrate = 0$ results in a systematic failure and this is expected. Reservoirs neurons are always silent and their states cannot be associated to an expected readout value. Depending on the spatial resolution used for sampling the trajectories, there is an adapted leak rate range that yields good performance.

Sequence complexity effects on consolidation

The stochastic gradient descent can occur because the activation values of X_{res} are reliable and everything happens as if the model had been exposed to the whole sequence. Given the visualization of the reservoir state induced by snippet replay, we now demonstrate that sequence learning is possible through snippet replay by running several simulations using data from rat traversals of different configurations from the behavioral dataset.

INSERT Figure 6 HERE

Figure 6 illustrates the effects of snippet size on sequence reconstruction performance. In general, longer snippets lead to better learning, though it is of interest to note the variability between performance on these 10 sequences of varying length and complexity from the rat behavior dataset. Different snippet sizes are possible and they are defined by the number of samples corresponding to a percentage $p \in]0, 1]$ of the total number of samples T_k of a k trajectory. Several snippets sizes are evaluated with the optimal parameters found in Basic Sequence learning. Figure 6 displays the Fréchet distance scores when the model learns ten sequences, based on random replay with different snippets sizes. The time budget parameter is fixed to $T = 184 * 100$, 100 times the size of the trajectory having the highest number of samples.

The maximum snippet size corresponds to the length of the shortest sequence itself. It is the particular case where the number of possible snippets is one. We are more interested by smaller snippet sizes that only represent partially the place-cells activations of the entire sequence because they model hippocampus place-cell replay during SWR.

The snippet size limits the size of the place-cell activation history (context) maintained by the reservoir. If the snippet size is shorter than the size of the context required for generating a trajectory, then the trajectory generated by the model will be different from the expected trajectory. This is not necessarily a bad property of snippet replay since it allows the model to generate novel trajectories that it can represent. We will continue to illustrate this property by examining other generated sequences extracted from the training set.

Fréchet distance scores of generated trajectories are clustered into two classes of trajectories by a standard K-means algorithm: the 'best' class aggregates trajectories having the smallest possible Fréchet distance score, and the 'others' class aggregates trajectories having the remaining scores. Table 3 shows for the trajectory found in configuration 34 and a limited range of snippets size, the population percentage of each class and its associated Fréchet distance score.

INSERT Table 3 HERE

For $snippet\ size\ =\ 9$, 91.2% of the generated trajectories have a Fréchet distance score of 0.037858. This is the smallest possible snippet size that allows acceptable generation performances for the trajectory found in Configuration 34, trial 2. $snippet\ size =\ 20$ is also an interesting point because even the 'others' class score has the same order of magnitude as the 'best' class score.

Sequences are globally generated with the best accuracy the model can reach for $snippet\ size\ =\ 10$. Snippet replay entails a performance drop compared to standard sequence learning paradigms but it is not relevant for navigation tasks. We observe that random snippet replay allows the model to align random subsequences of a trajectory and consolidate them; to break the long range dependencies by using a short snippet size and to generate any trajectory the model can represent. Now we will see the advantages of this capability.

Analysis of different degrees of reverse replay

In the current experiment, we examine the effects of forward and reverse replay during the sequence learning process. At the same time, we investigate the effects of forward and reverse reward propagation during the creation of the hippocampal replay model. We first validate that a random replay containing reversed snippets allows the model to generate sequences in both directions. One hundred instances of the model are exposed to a random replay of snippets having size 10 during a reverse replay stage that is evaluated over the following parameters ranges: Reverse rate varying from 0 to 1 with increments of 0.1.

We examine the train-test direction pairs forward-forward, forward-reverse, reverse-forward and reverse-reverse as illustrated in Figure 14, as we vary the proportion of reverse replay. For each corresponding condition (Cartesian product of hippocampal reward propagation reverse rate, and replay direction reverse rate), the model is successively reset to its initial state and trained.

INSERT Figure 7 HERE

As shown in Figure 7, the model is able to generate the ABCDE sequence in forward and backward direction and best performances are achieved when about half of the snippets of ABCDE are entirely reversely replayed. The same conclusion applies for sequence EDCBA. At this point, the effect of reverse replay is to have virtually a forward random replay of a given trajectory in forward direction (as experienced by the agent) and the same trajectory experienced in the reverse direction.

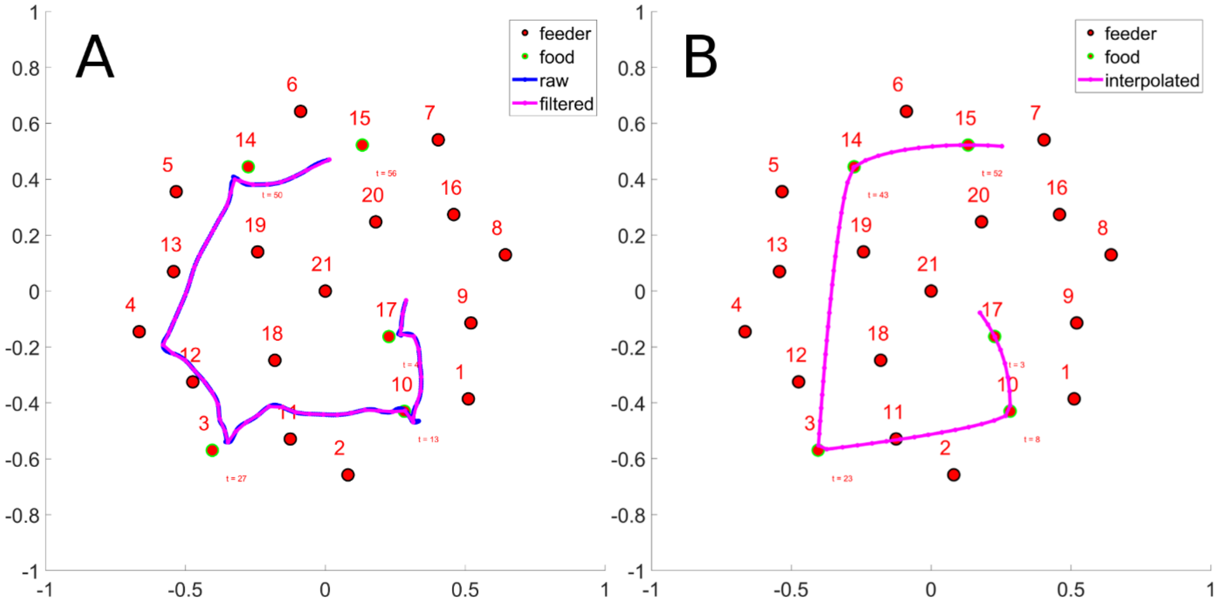

Figure 1: Example Rat trajectory. A. One trial of the rat traversal of configuration 84. B. Idealized version of this trajectory that can be compared with trajectories generated by the model.

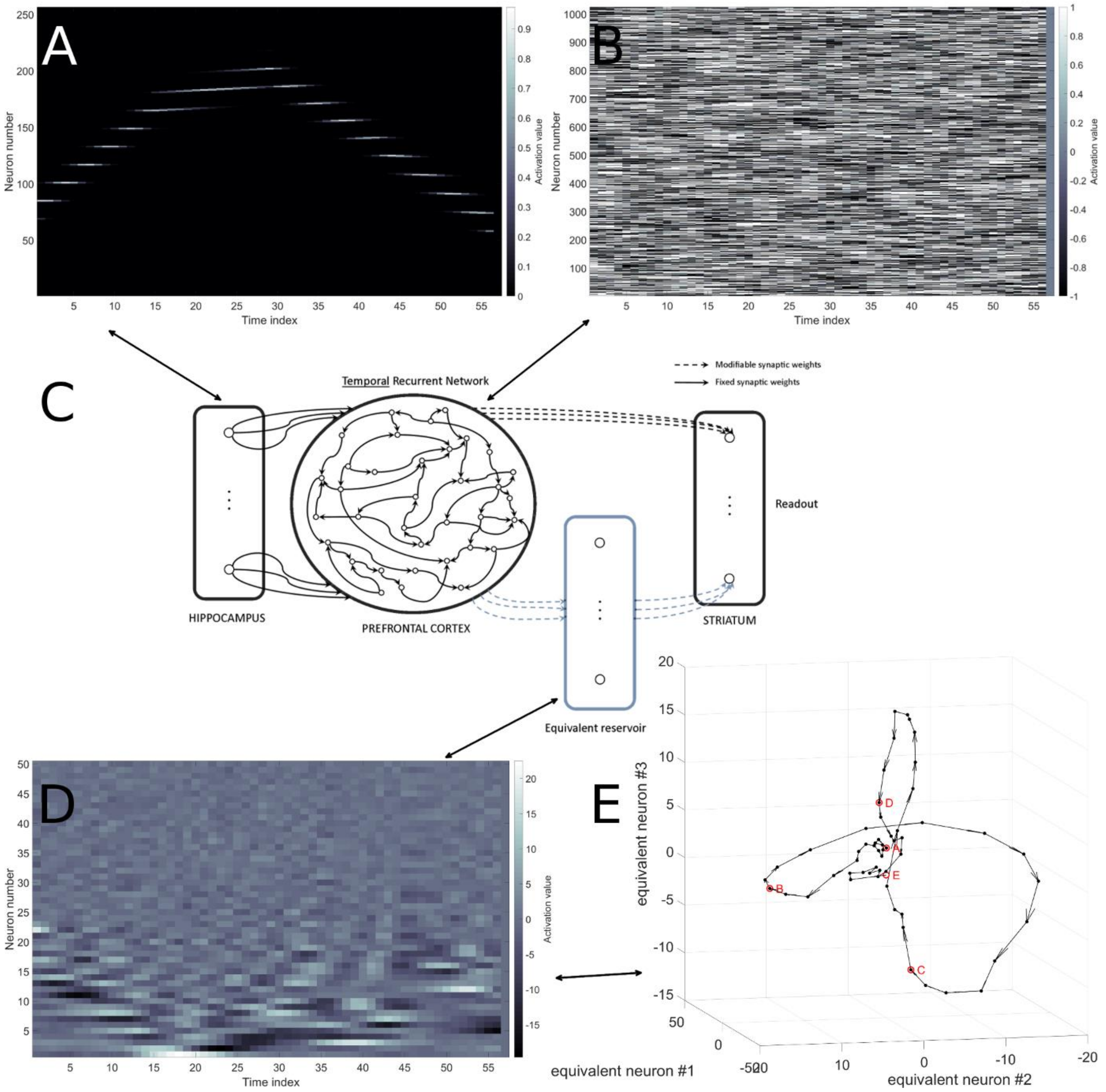

Figure 2: Internal Representations in the reservoir. Panel A shows the raster of simulated hippocampus place-cell neurons activation over time. Panel B represents the raster of the reservoir's neurons. Panel C depicts the hippocampus, prefrontal cortex and striatum as we modelled them and features a supplementary artificial neuron group called equivalent reservoir whose raster is represented in panel D. An equivalent reservoir neuron represents in fact a principal component, as computed by the PCA algorithm. Panel E represents the raster observed in panel D as a 3D trajectory resulting from the 3 first principal components.

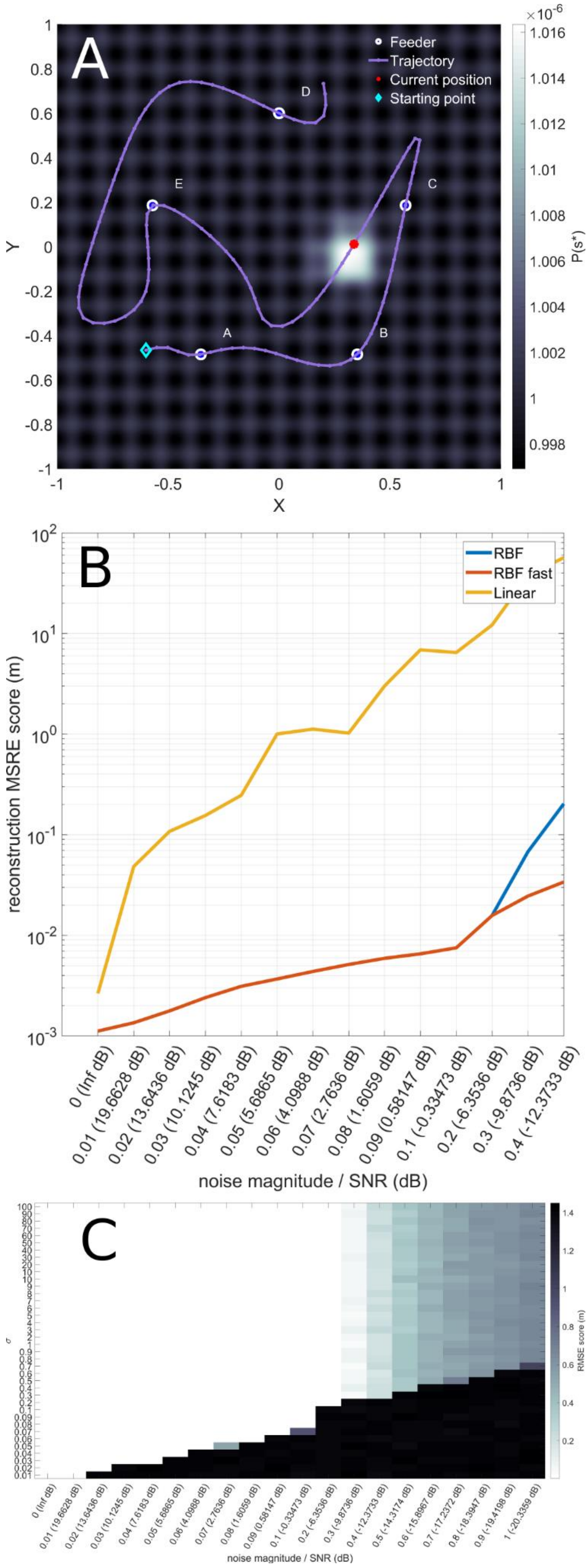
A
Feeder
Trajectory
Current position
Starting point
A
B
C
D
E
X
Y
P(s*)
×10^-6
B
RBF
RBF fast
Linear
reconstruction MSRE score (m)
noise magnitude / SNR (dB)
0 (Inf dB)
0.01 (19.6628 dB)
0.02 (13.6436 dB)
0.03 (10.1245 dB)
0.04 (7.6183 dB)
0.05 (5.6865 dB)
0.06 (4.0988 dB)
0.07 (2.7636 dB)
0.08 (1.6059 dB)
0.09 (0.58147 dB)
0.1 (-0.33473 dB)
0.2 (-6.3536 dB)
0.3 (-9.8736 dB)
0.4 (-12.3733 dB)
C
σ
RMSE score (m)
noise magnitude / SNR (dB)

Figure 3: Spatial Filter. Panel A represents the location probability map computed by our Bayesian reconstruction method and result from a Gaussian kernel with $\sigma = 10$ applied to the norm between the place-cell activation of each point of the map and the predicted next place-cell activation pattern computed by the reservoir model. The trajectory ABCED is overlaid on the map and the current position corresponding to the input activation pattern is shown in red. Panel B depicts the reconstruction error in function of the noise magnitude/signal to noise ratio of the linear direct model (Average of the place-cells centers weighted by the normalized mean firing rate of the place –cells), our radial basis function method (the Gaussian kernel is a radial basis function) computed over the full map and a faster version of our method where the computation is limited to a circle surrounding the current position. Panel C shows an extensive evaluation of the firing rate map-based kernel decoding algorithm. Noise varies from 0% to 100% of mean firing rate and sigma varies from 0.01 to 100 in a semi logarithmic manner.

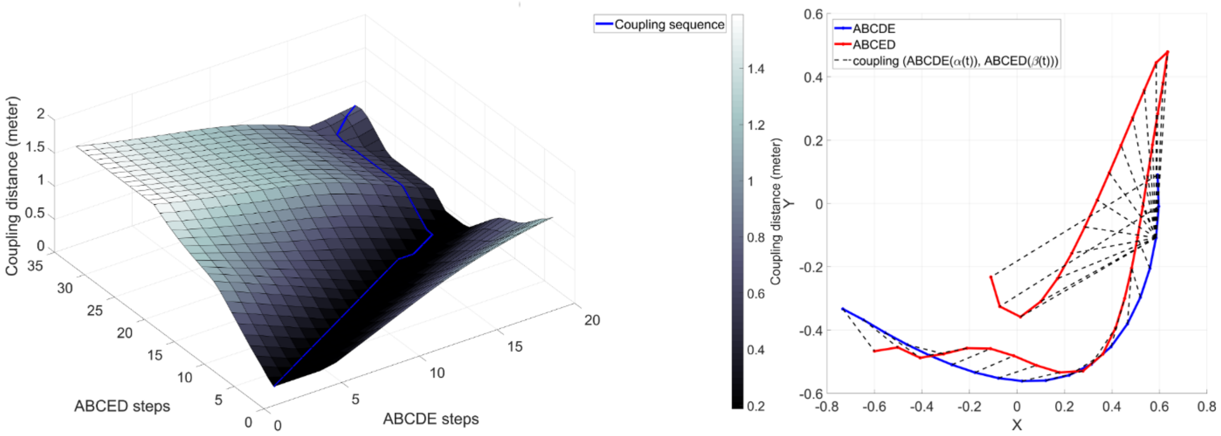

A B

Figure 4: Fréchet Distance. Panel A shows the free space diagram used during the computation of the Fréchet distance between ABCED and ABCDE trajectories. X and Y axis represent the discrete steps between points of ABCED and ABCDE trajectories. Z axis relates in meter the coupling distance between two points. The coupling sequence is represented with a blue line following the optimal alignment between the two trajectories (i.e. sequences of points). Panel B represents the paired points of ABCDE and ABCED trajectories. During the common prefix ABC, curves are relatively similar and the paired points are located close to each other. This is related by the diagonal part of the coupling sequence in panel A. Then the diverging parts of ABCDE and ABCED are still paired but we can clearly observe a one-to-many relationship, near point (0.6,-0.1) and the free space diagram relates it by variations along the ABCED steps axis and no variation along the ABCDE steps axis.

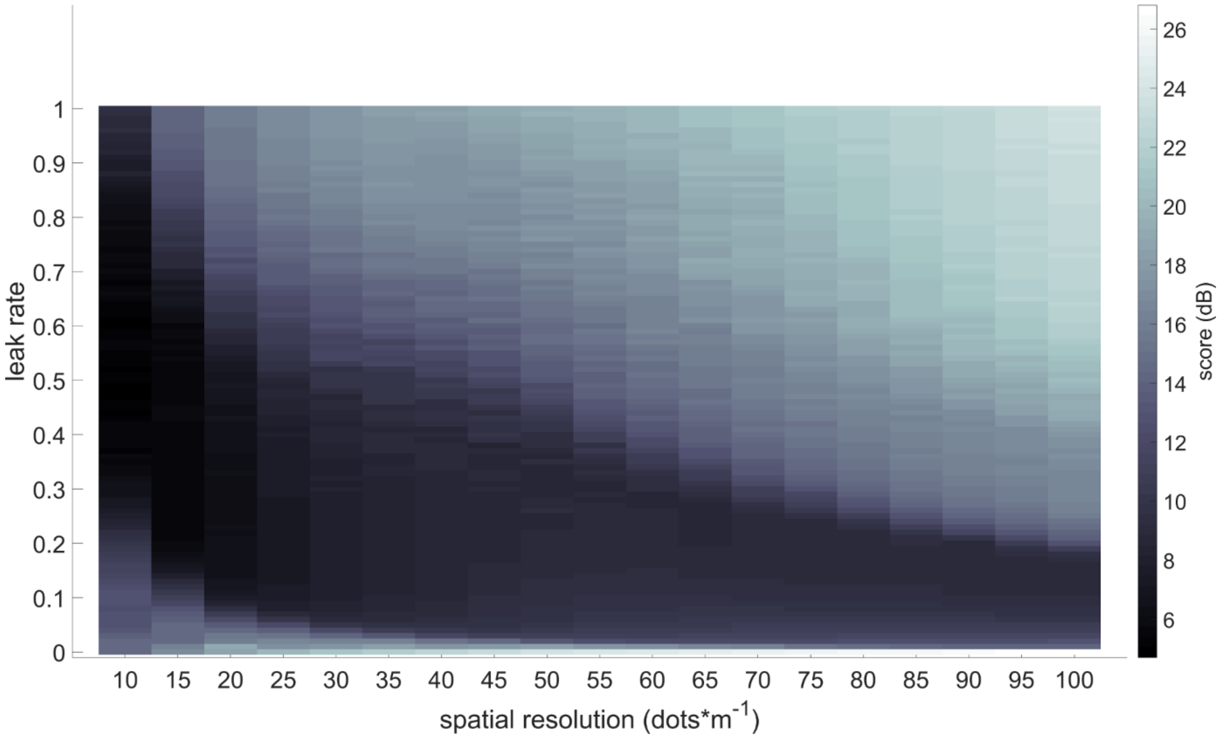

Figure 5: Spatial resolution vs. reservoir neuron leak rate. As the spatial resolution increases, the number of discrete points that describe a sequence increases. We observe that as this occurs, the corresponding leak rate decreases. This indicates the relation between sequence length and local temporal memory implemented at the individual neuronal level.

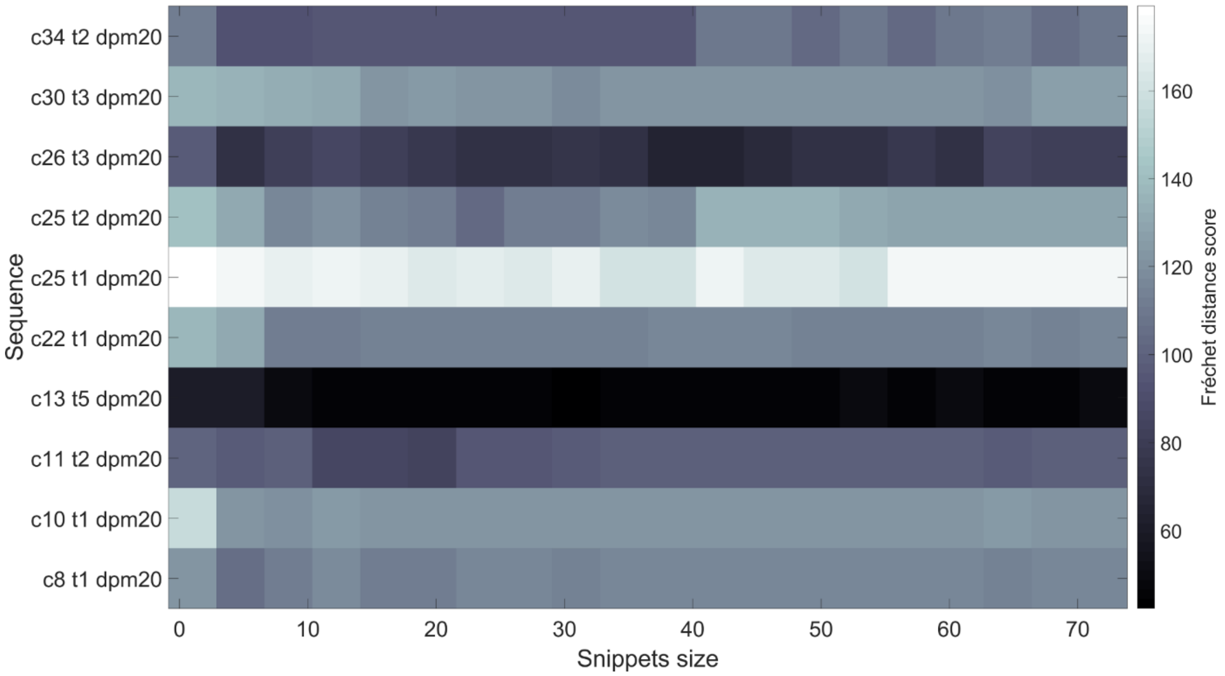

Figure 6: Effects of snippet size on sequence learning for sequences of varying difficulty. Sequences from ten configurations of the rat TSP data set were used with snippets of different sizes varying from 0 to 70. We observe that for most sequences, error is reduced as snippet size increases.

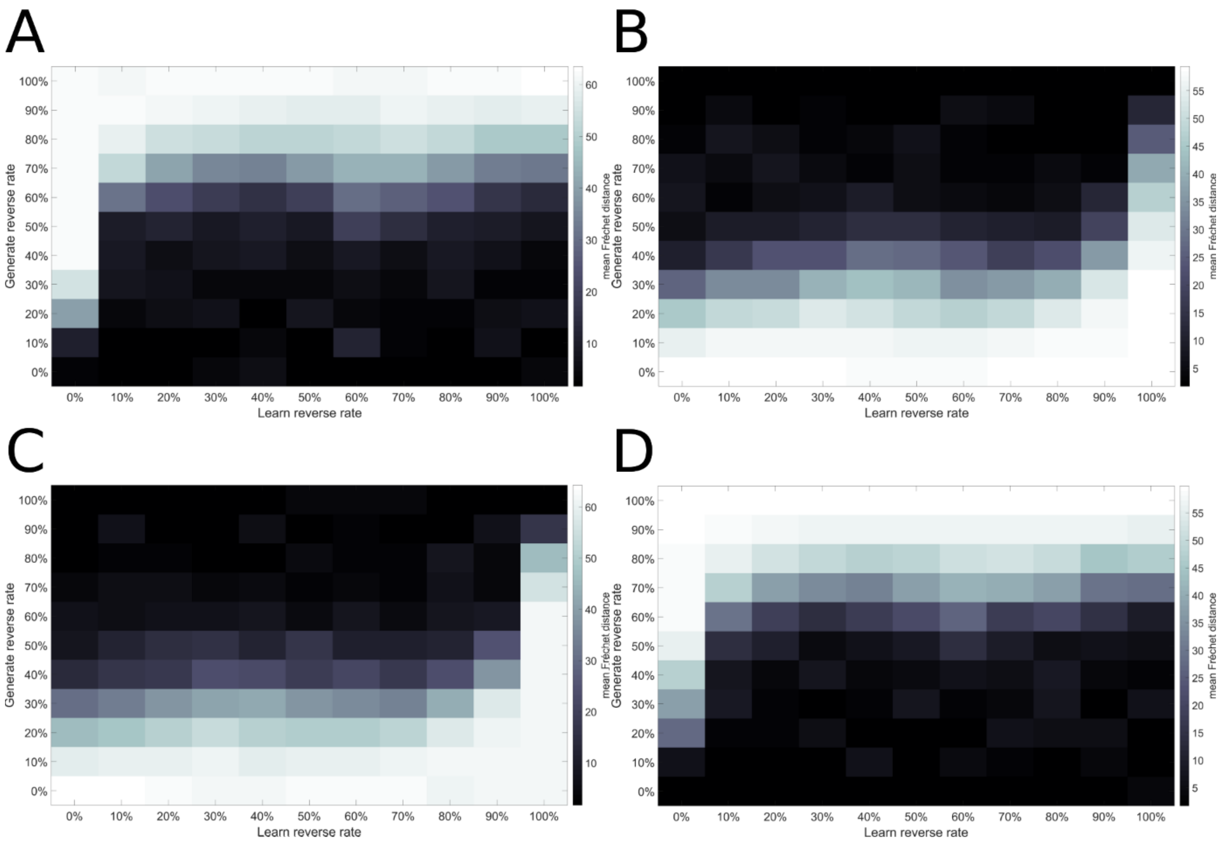

Figure 7: Effects of reverse replay and initial sequence direction. When a sequence is learned and tested in the same direction, introduction of reverse replay gradually impairs performance (panels A and D). When training and testing direction are reversed, then the introduction of reverse replay gradually improves performance. Interestingly, a mixture of forward and reverse replay leads to a general compromise for all situations.

Clusters of conditions number		Edge direction		
		**Forward**	**Backward**	**Both**
Required number of trial(s)	**1**	∅	84	41,43,53,78,84
	**2**	8,25,33,41,44,48,52,70,78	11	8,11,25,29,32,33,38,40,44,52,56,70,82
	**3**	97	58,80	48,80,97
**Total number of configurations**		10	4	21

Table 1: Classification of rat behavioral configurations by direction in which executed trajectories relate to desired trajectory.

**Solution number**	**in_scale**	**leak_rate**	**rec_scale**	**score**
1	'6.379720'	'0.494997'	'6.553708'	1,1879078
2	'5.212805'	'0.481997'	'5.542781'	1,1900330
3	'6.429717'	'0.485997'	'6.553708'	1,1994574
4	'5.189806'	'0.480997'	'5.542781'	1,2003332
5	'5.404791'	'0.483997'	'5.542781'	1,2313991
6	'5.256802'	'0.508997'	'5.542781'	1,2524340
7	'9.084041'	'0.445998'	'8.420773'	1,2691536
8	'5.211805'	'0.495997'	'5.542781'	1,2712017
9	'6.322724'	'0.492997'	'6.952679'	1,2836924
10	'8.297723'	'0.459998'	'5.542781'	1,3022404

Table 2: summary of the 10 parameter sets optimized by the parallel simulated annealing algorithm. Column in_scale represents the scaling constant applied to the feedforward connectivity matrix $\boldsymbol{W_{in}}$, rec_scale represents the scaling constant applied to the recurrent connectivity matrix $\boldsymbol{W_{rec}}$, normalized by $\frac{1}{\sqrt{N}}$ and ensuring an approximately constant spectral radius of $\boldsymbol{\rho} \approx \mathbf{3.87}$. Column leak_rate represents different values of the leak rate of the reservoir neurons and solution number, the rank of the parameter set sorted in increasing order

Snippets size		1	5	9	12	16	20	23	27	31
best	Score	0.53583	0.13909	**0.037858**	0.037005	0.021245	0.015277	0.014615	0.013871	0.020241
	Population %	29.9	52.1	**91.2**	98.6	98.2	94.9	83.7	77.8	99
others	Score	0.98145	0.92174	0.73686	1.0041	0.87086	0.029701	0.025379	0.022013	0.95653
	Population %	70.1	47.9	8.8	1.4	1.8	5.1	16.3	22.2	1

Table 3: Performance (revealed by Fréchet distance to desired trajectory) as a function of snippet size.

References:

Cortes, C. and V. Vapnik (1995). "Support-vector networks." Machine learning **20**(3): 273-297.

Eiter, T. and H. Mannila (1994). "Computing discrete Frechet distance." Tech. Report CD-TR 94/64, Information Systems Department, Technical University of Vienna.

Lukosevicius, M. (2012). A practical guide to applying echo state networks. Neural networks: tricks of the trade, Springer: 659-686.

Ram, D. J., T. H. Sreenivas and K. G. Subramaniam (1996). "Parallel simulated annealing algorithms." Journal of parallel and distributed computing **37**(2): 207-212.

Rigotti, M., O. Barak, M. R. Warden, X.-J. Wang, N. D. Daw, E. K. Miller and S. Fusi (2013). "The importance of mixed selectivity in complex cognitive tasks." Nature.

Zhang, K., I. Ginzburg, B. L. McNaughton and T. J. Sejnowski (1998). "Interpreting neuronal population activity by reconstruction: unified framework with application to hippocampal place cells." Journal of neurophysiology **79**(2): 1017-1044.
