# Supplementary figures and images for: Reservoir computing model of prefrontal cortex creates novel combinations of previous navigation sequences from hippocampal place-cell replay with spatial reward propagation
Source: PLoS Comput Biol. 2019 Jul 15;15(7):e1006624. doi: 10.1371/journal.pcbi.1006624 (PMC6668845; doi:10.1371/journal.pcbi.1006624)

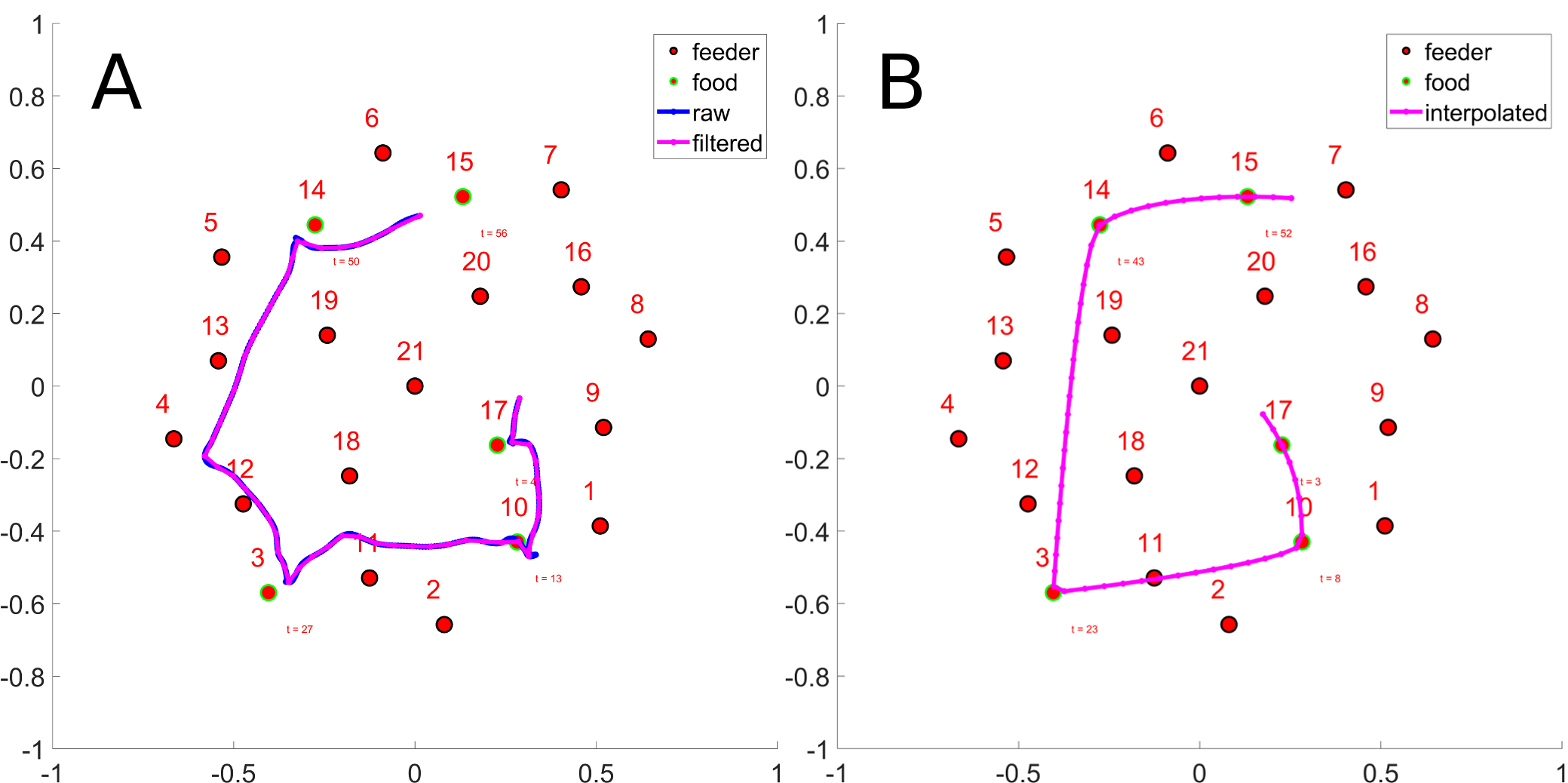

Supplement: S1 Fig — (TIF) [file pcbi.1006624.s002.tif]

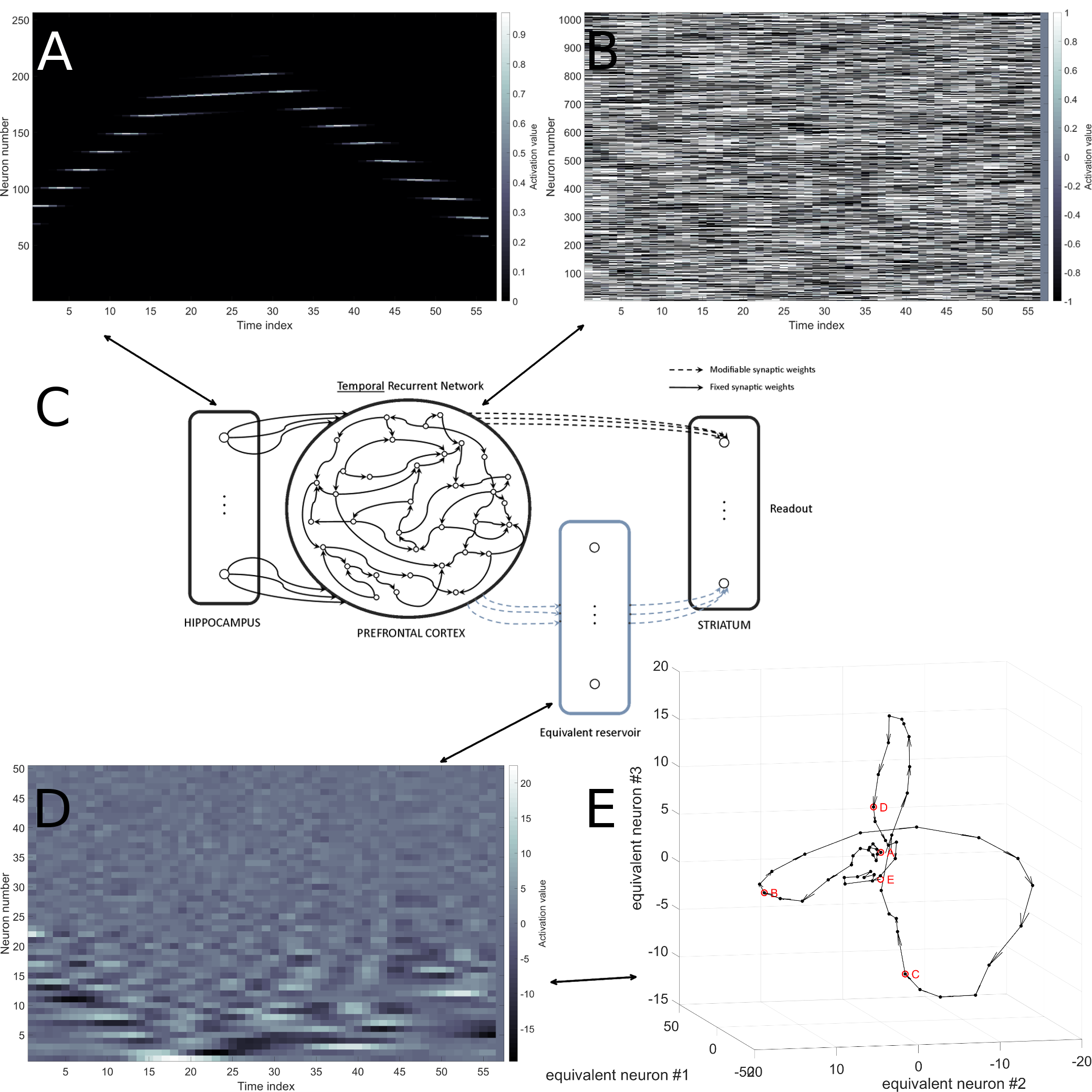

Supplement: S2 Fig — (TIF) [file pcbi.1006624.s003.tif]

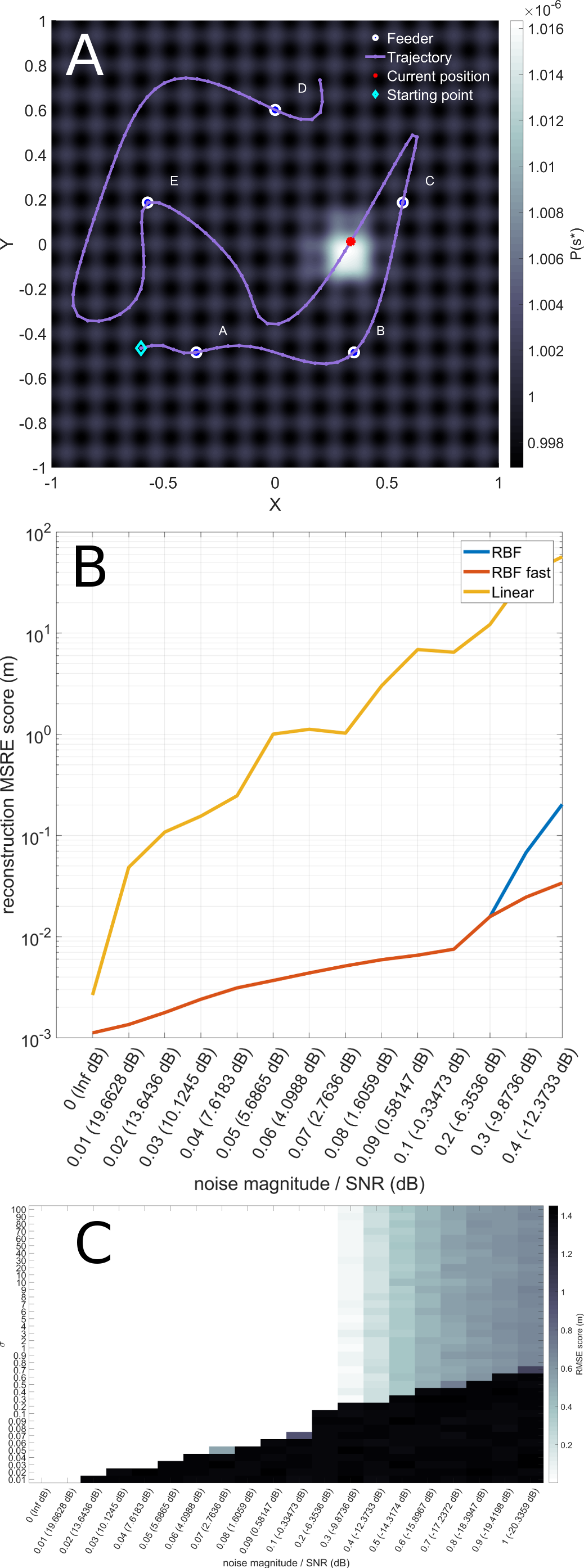

Supplement: S3 Fig — (TIF) [file pcbi.1006624.s004.tif]

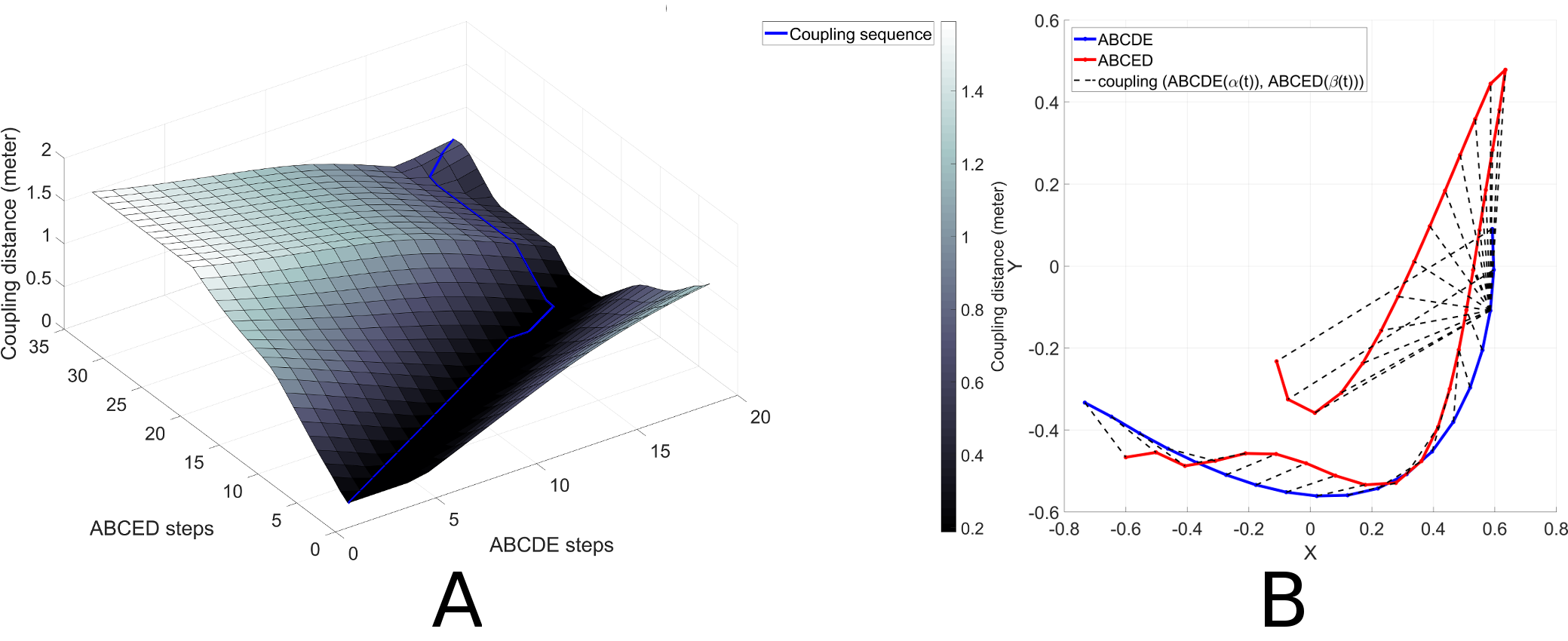

Supplement: S4 Fig — (TIF) [file pcbi.1006624.s005.tif]

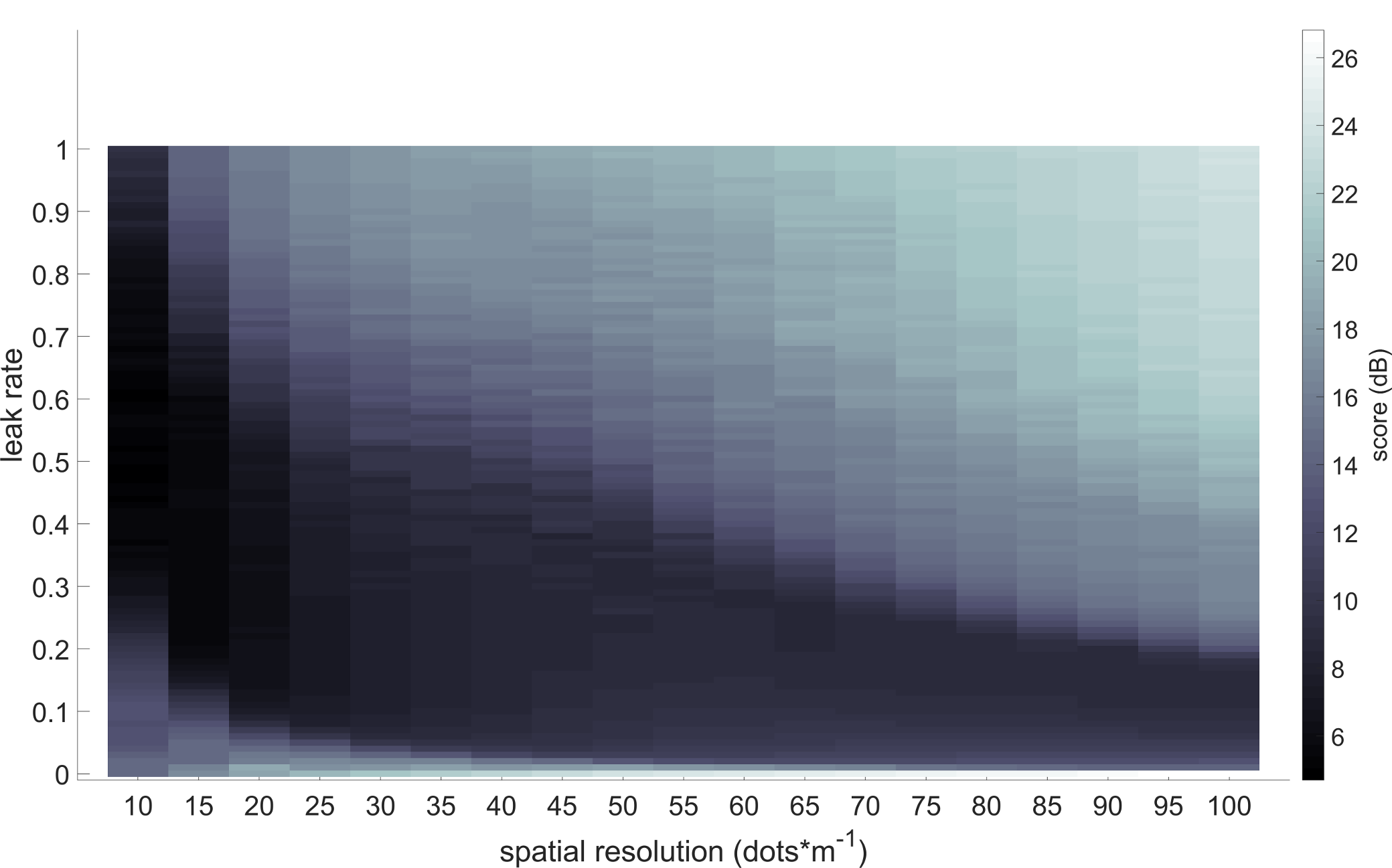

Supplement: S5 Fig — (TIF) [file pcbi.1006624.s006.tif]

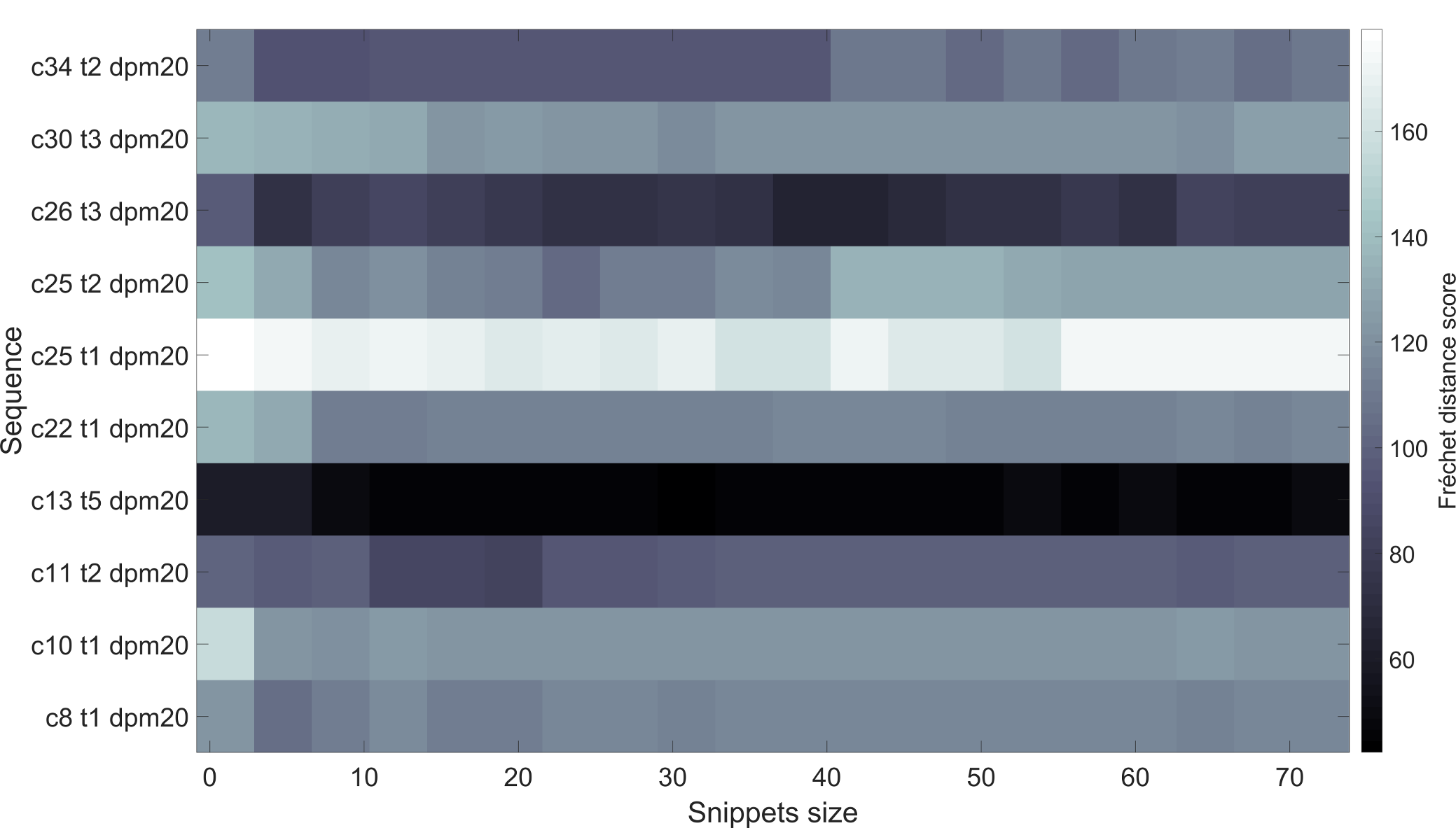

Supplement: S6 Fig — (TIF) [file pcbi.1006624.s007.tif]

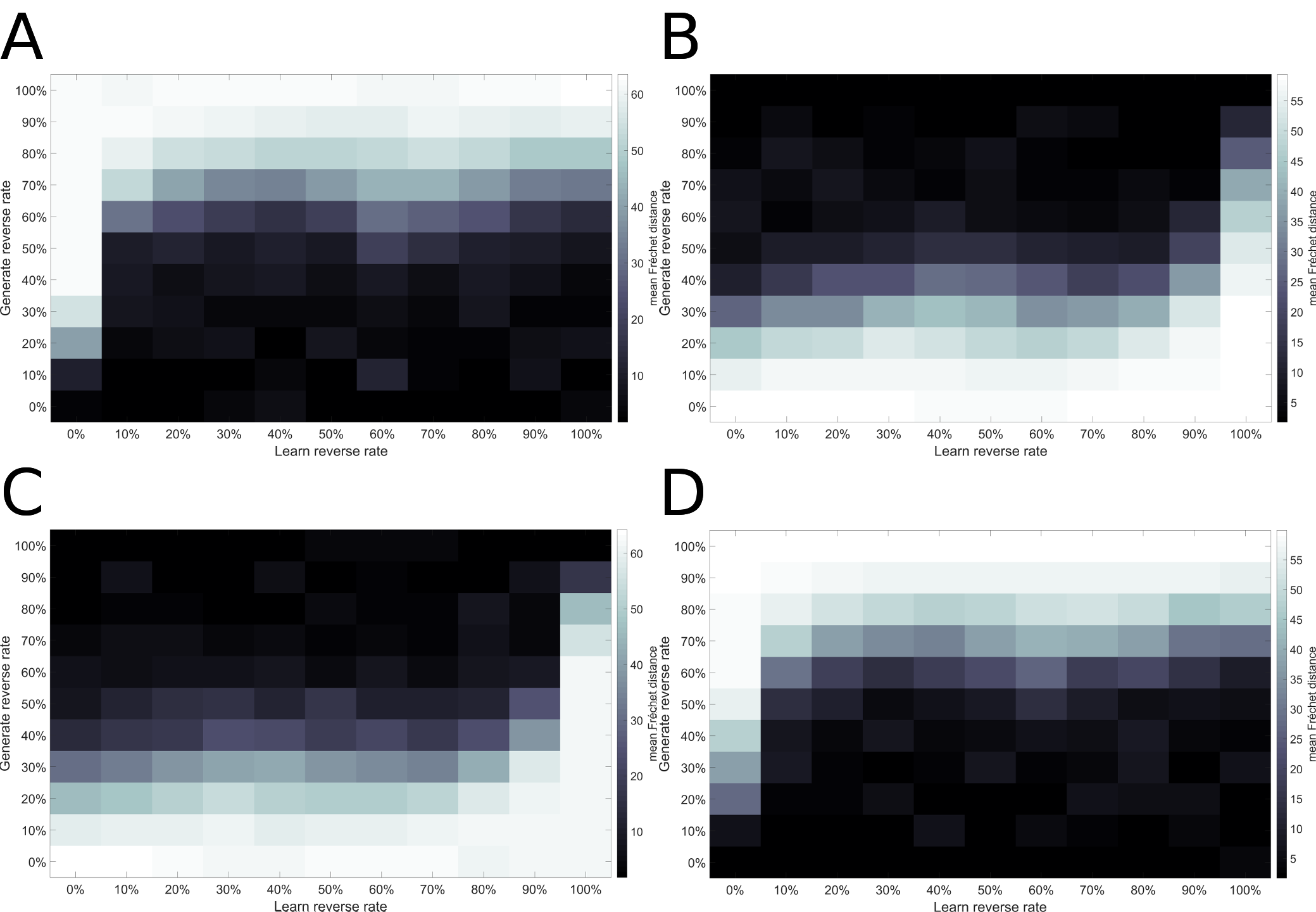

Supplement: S7 Fig — (TIF) [file pcbi.1006624.s008.tif]
