# Supplementary material for: Reservoir computing model of prefrontal cortex creates novel combinations of previous navigation sequences from hippocampal place-cell replay with spatial reward propagation
Source: PLoS Comput Biol. 2019 Jul 15;15(7):e1006624. doi: 10.1371/journal.pcbi.1006624 (PMC6668845; doi:10.1371/journal.pcbi.1006624)
Supplement: S1 Table — (DOCX) [file pcbi.1006624.s009.docx]

Cazin S1 - Table

| Clusters of conditions number | | Edge direction | | |
| --- | --- | --- | --- | --- |
|  |  | **Forward** | **Backward** | **Both** |
| Required number of trial(s) | **1** | $\emptyset$ | 84 | 41,43,53,78,84 |
|  | **2** | 8,25,33,41,44,48,52,70,78 | 11 | 8,11,25,29,32,33,38,40,44,52,56,70,82 |
|  | **3** | 97 | 58,80 | 48,80,97 |
| **Total number of configurations** | | 10 | 4 | 21 |

Table 1: Classification of rat behavioral configurations by direction in which executed trajectories relate to desired trajectory.
