# Supplementary material for: Reservoir computing model of prefrontal cortex creates novel combinations of previous navigation sequences from hippocampal place-cell replay with spatial reward propagation
Source: PLoS Comput Biol. 2019 Jul 15;15(7):e1006624. doi: 10.1371/journal.pcbi.1006624 (PMC6668845; doi:10.1371/journal.pcbi.1006624)
Supplement: S2 Table — (DOCX) [file pcbi.1006624.s010.docx]

Cazin S2 – Table

| **Solution number** | **in_scale** | **leak_rate** | **rec_scale** | **score** |
| --- | --- | --- | --- | --- |
| 1 | '6.379720' | '0.494997' | '6.553708' | 1,1879078 |
| 2 | '5.212805' | '0.481997' | '5.542781' | 1,1900330 |
| 3 | '6.429717' | '0.485997' | '6.553708' | 1,1994574 |
| 4 | '5.189806' | '0.480997' | '5.542781' | 1,2003332 |
| 5 | '5.404791' | '0.483997' | '5.542781' | 1,2313991 |
| 6 | '5.256802' | '0.508997' | '5.542781' | 1,2524340 |
| 7 | '9.084041' | '0.445998' | '8.420773' | 1,2691536 |
| 8 | '5.211805' | '0.495997' | '5.542781' | 1,2712017 |
| 9 | '6.322724' | '0.492997' | '6.952679' | 1,2836924 |
| 10 | '8.297723' | '0.459998' | '5.542781' | 1,3022404 |

Table 2: summary of the 10 parameter sets optimized by the parallel simulated annealing algorithm. Column in_scale represents the scaling constant applied to the feedforward connectivity matrix $\boldsymbol{W}_{\boldsymbol{in}}$, rec_scale represents the scaling constant applied to the recurrent connectivity matrix $\boldsymbol{W}_{\boldsymbol{rec}}$, normalized by $\frac{\boldsymbol{1}}{\sqrt{\boldsymbol{N}}}$ and ensuring an approximately constant spectral radius of $\boldsymbol{\rho\approx3.87}$. Column leak_rate represents different values of the leak rate of the reservoir neurons and solution number, the rank of the parameter set sorted in increasing order
