# Supplementary material for: Reservoir computing model of prefrontal cortex creates novel combinations of previous navigation sequences from hippocampal place-cell replay with spatial reward propagation
Source: PLoS Comput Biol. 2019 Jul 15;15(7):e1006624. doi: 10.1371/journal.pcbi.1006624 (PMC6668845; doi:10.1371/journal.pcbi.1006624)
Supplement: S3 Table — (DOCX) [file pcbi.1006624.s011.docx]

Cazin S3 – Table

| Snippets size | | 1 | 5 | 9 | 12 | 16 | 20 | 23 | 27 | 31 |
| --- | --- | --- | --- | --- | --- | --- | --- | --- | --- | --- |
| best | Score | 0.53583 | 0.13909 | **0.037858** | 0.037005 | 0.021245 | 0.015277 | 0.014615 | 0.013871 | 0.020241 |
|  | Population % | 29.9 | 52.1 | **91.2** | 98.6 | 98.2 | 94.9 | 83.7 | 77.8 | 99 |
| others | Score | 0.98145 | 0.92174 | 0.73686 | 1.0041 | 0.87086 | 0.029701 | 0.025379 | 0.022013 | 0.95653 |
|  | Population % | 70.1 | 47.9 | 8.8 | 1.4 | 1.8 | 5.1 | 16.3 | 22.2 | 1 |

Table 3: Performance (revealed by Fréchet distance to desired trajectory) as a function of snippet size.
