# Supplementary material for: Reservoir computing model of prefrontal cortex creates novel combinations of previous navigation sequences from hippocampal place-cell replay with spatial reward propagation
Source: PLoS Comput Biol. 2019 Jul 15;15(7):e1006624. doi: 10.1371/journal.pcbi.1006624 (PMC6668845; doi:10.1371/journal.pcbi.1006624)
Supplement: S1 Data — (ZIP) [file CRCNS_2016.pdf › CRCNS_2016.pdf]

Prefrontal cortex reservoir network learns to create novel efficient navigation sequences by concatenating place-cell snippets replayed in hippocampus

52

N.CAZIN[1,2], M.LLOFRIU ALONSO[3], P.SCLEIDOROVICH CHIODI[3], T.PELC[4], A.WEITZENFELD[3], J-M.FELLOUS[4], P.F.DOMINEY[1,2]

[1]INSERM U1208/SBRI, Bron, France; [2]Univ. de Lyon, Univ. Lyon 1, Lyon, France; [3]Computer Scie. and Engin., Univ. of South Florida, Tampa, FL; [4]Psychology, Univ. of Arizona, Tucson, AZ

Introduction

Spatial navigation in the rat involves the **replay** of **place cell subsequences** (snippets) during awake and sleep states in the **hippocampus** during sharp-wave-ripple (SPWR) events ([1], [2]). We focus on the role of replay during the **awake** state, as the animal generates **increasingly efficient trajectories** between reward sites, across multiple trials. This trend toward optimal solutions is referred to as the **Traveling Salesman Problem** (TSP). We hypothesize that **snippet replay** allows recurrent dynamics in prefrontal cortex (PFC) to **consolidate** snippet representations to create novel efficient sequences in TSP, by **rejecting** training sequences that are **less robustly coded** in the input.

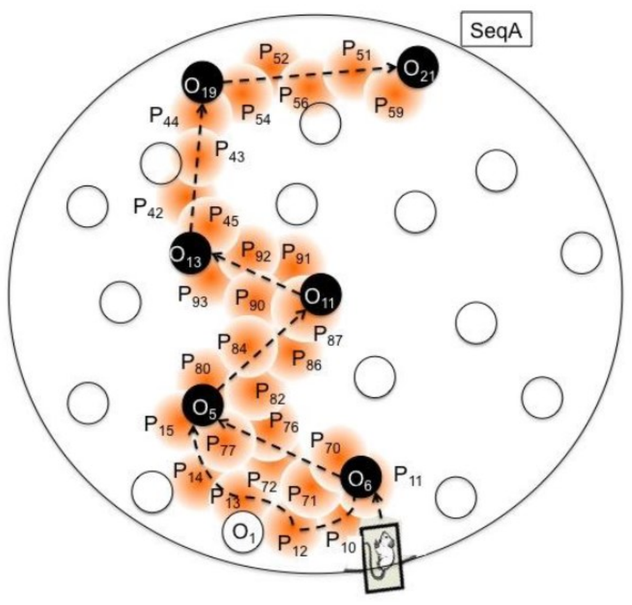

Temporal Recurrent Network for sequence learning

Prefrontal cortex is modeled as an instantiation of the **reservoir computing** paradigm called the Temporal Recurrent Network (TRN) [3], a computational metaphor of the **cortico-cortical** (reservoir) and **corticostriatal** (readout) circuits. Inputs excite a pool of neurons having fixed **recurrent** connections, generating high dimensional dynamics through **reverberation**. The principle is straightforward:

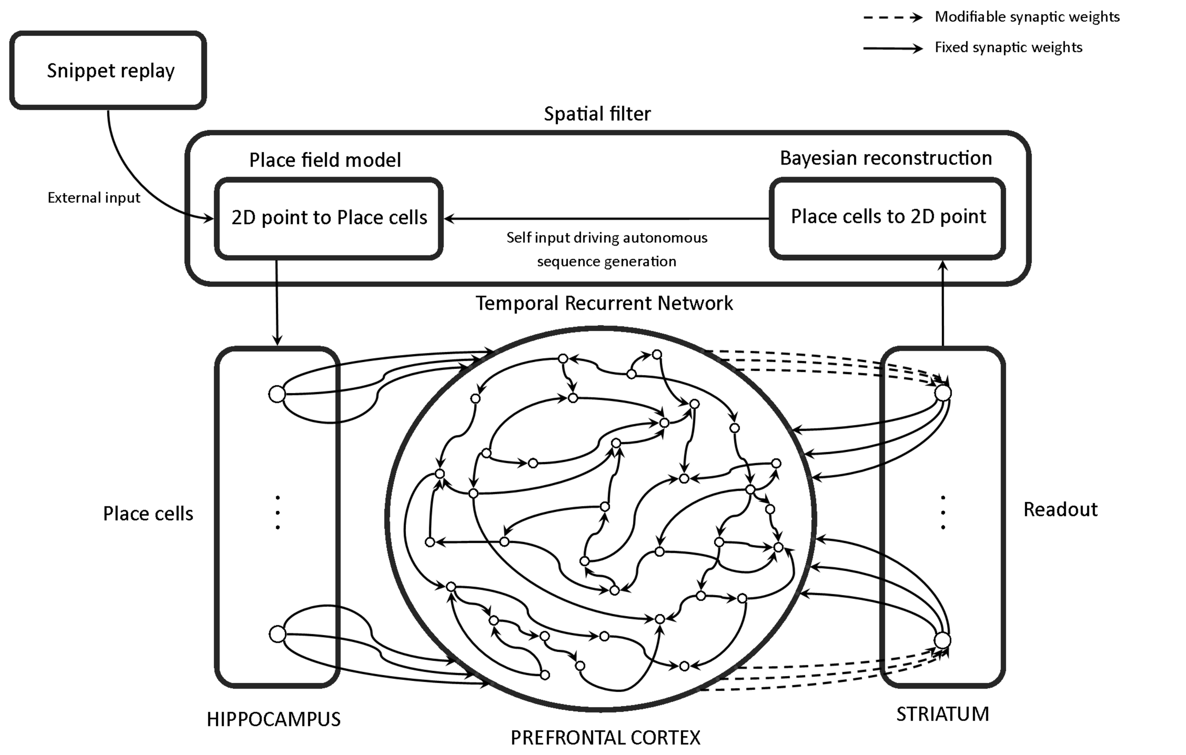

1. Input neurons (hippocampal place cells - HPCs) fire and induce a **high dimensional time dependent activity** pattern on the PFC reservoir's neurons
2. The desired output (next location coded HPCs) is **reconstructed** in readout neurons (striatum) by reading the reservoir's neurons and correcting the synaptic weights in order to **minimize** the **quadratic error** between the actual output and the expected output.
3. The predicted output feeds back (through a spatial filter) as self input driving autonomous sequence generation.

Snippet replay generates training input

Snippet replay is modeled as generation of **random subsequences** of **place cell** firing patterns that occurred during previous trials. **Snippet length** (i.e. duration) is **fixed** and a **limited time budget** is allocated to snippet replay. Snippets are generated between random pairs of reward sites in previous trajectories. **Shorter paths** between rewarded sites are **more robustly coded** due to greater snippet overlap. Here is an example of a snippet of the ABCED sequence. It lasts 5 simulation ticks and both spatial and place-cell representation are shown.

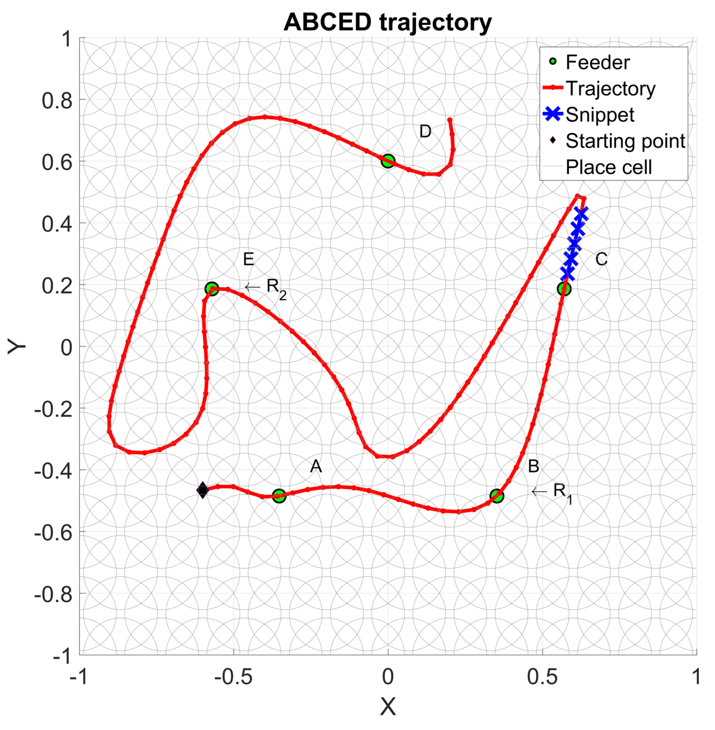

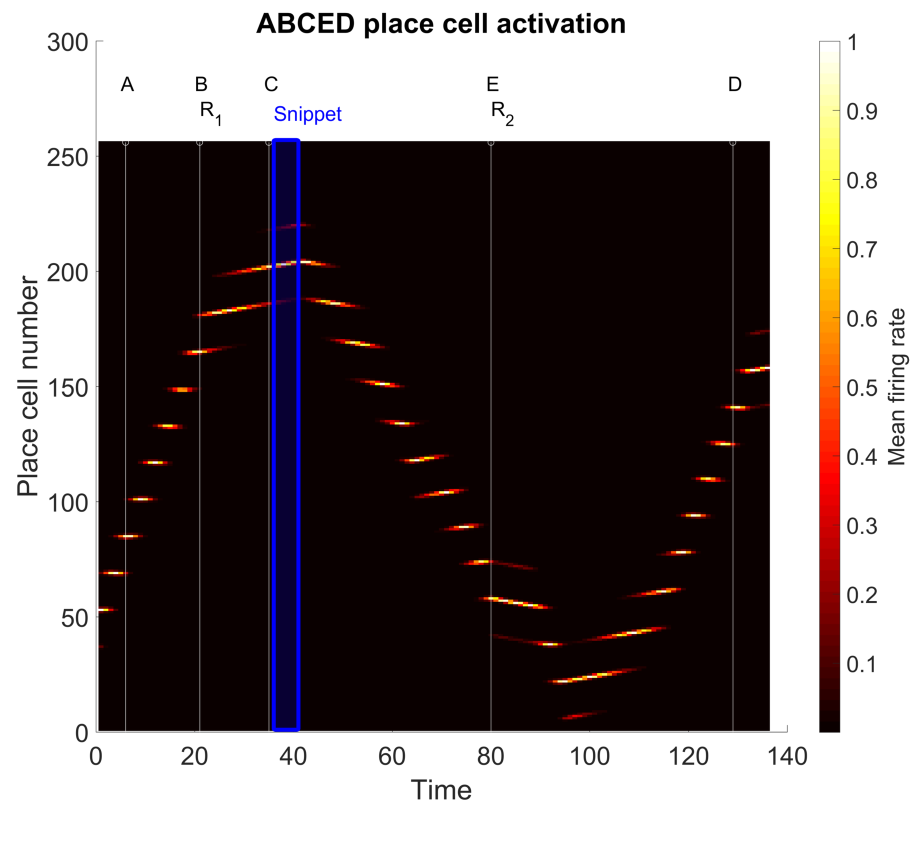

Spatial filter (reconstruction) during autonomous sequence generation

During autonomous sequence generation, HPC readouts code the next location in the sequence and feed-back to the input. To reduce noise in this feedback signal, we **reconstruct** the 2D location from a weighted average of **location hypothesis** carried by HPC [4] and then generate a corresponding HPC activation pattern coding this location to be **reinjected** as input.

We propose the following method : Given the firing rate maps of 256 place-cells $(A_i)_{1 <= i <= 256}$, and a location s, place-cell responses a_i=$A_i(s)$ encode partial information about location *s*. Applying a **gaussian kernel** to A_i, centered on a_i will result in a map representing the probability of a 2D point $s*$ to generate the activation a_i.

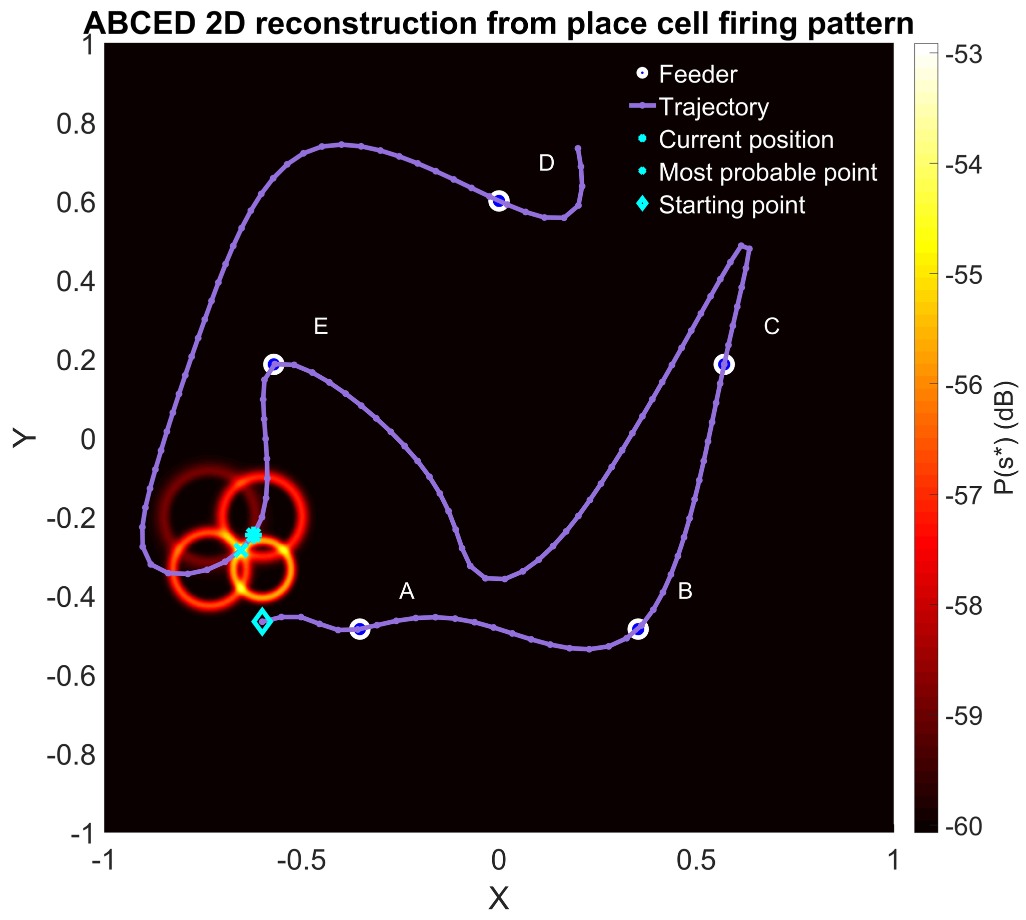

This map is noted $P(s*|a_i)$ and will take values close to 1 for a_i values close to $A_i(s)$. Values close to 0 will be associated with points not related to $A_i(s)$. Finally, the map that **estimates** the **position** of the possible points $s*$ that generated the place-cell activation pattern (a_i) is given by:

$$P(s*) = \sum_{i=1}^{N} P(s*|a_i)P(a_i) \qquad P(s*|a_i) = K * e^{-\frac{||A_i(s*)-a_i||^2}{\sigma^2}}$$

Given a **prediction** of a **place cell** activation pattern and its associated **location probability** map, **computing** the most probable location and generating an HPC activation pattern coding this location results in significant **reduction** of accumulated **noise** in the HPC **feedback** signal.

Trajectories are consolidated from random replay

TRN reservoir activation values over time can be represented as **trajectories** by using the principal component analysis (**PCA**). **Similar stimuli** are coded in **similar trajectories** in the reservoir's state space. The context provided by the recurrent connections induces slight differences in the encoding of similar sequences that have different preambles. A certain **time** is required for **fading** of this context. For a given context length, a **sufficient overlap** between snippets will ensure a **proper consolidation** of the whole trajectory. **Insufficient snippet overlap** results in **consolidation failure** (desired for non-optimal sequences).

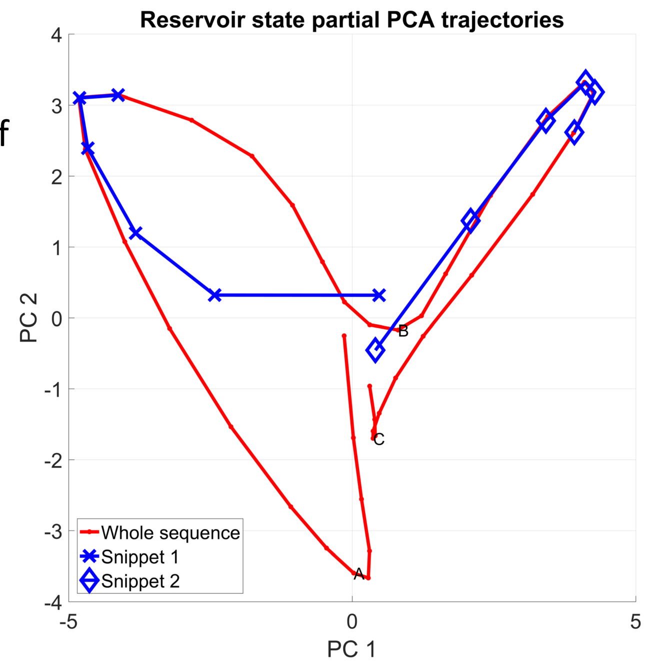

Longer paths are rejected

Two sequences joining sites ABE and ABCDE are replayed. Spatial distribution will be **sparse** between two rewarded sites linked by a **long** path and will be **denser** for a **shorter** path. The higher density yields greater overlap between snippets which favors them in competition during the learning process. Thus, for the same time of exposure to random replay, short paths between rewards will tend to be favored by greater snippet overlap.

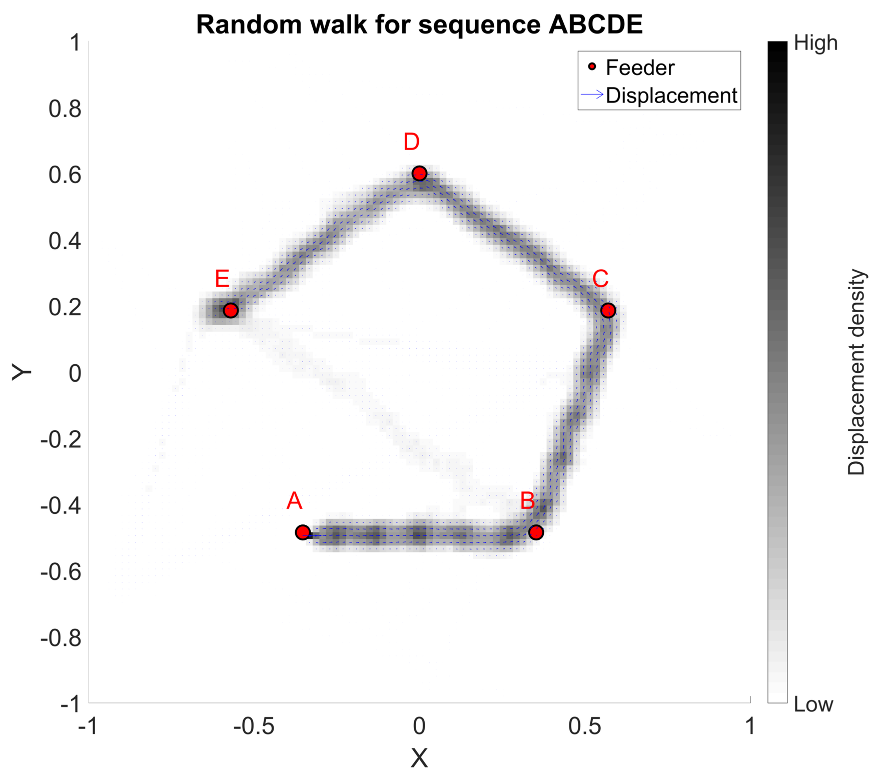

Condition \ Test	Primed with ABE	Primed with ABCDE
No intermediate reward	ABE generated	ABE generated
Intermediate Reward	ABCDE generated	ABCDE generated

Novel efficient navigation sequence is created

Three different trajectories (ABCED, BACDE, EBCDA) linking five reward sites contain parts of the **target sequence** ABCDE.

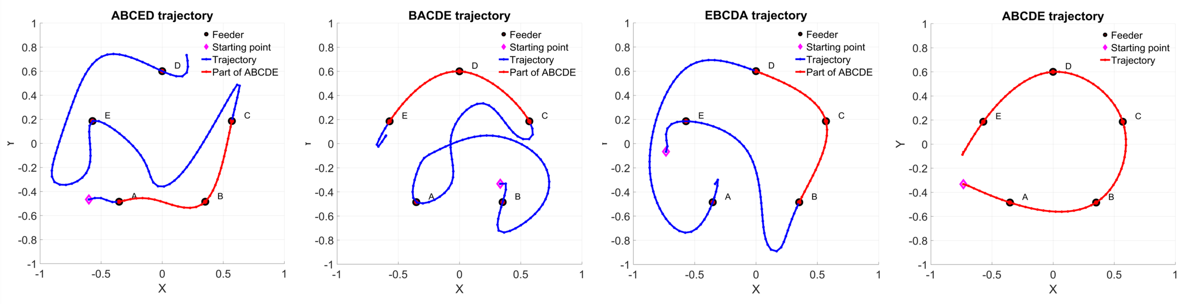

10 instances of the model are trained only with random snippets of sequences ABCED, BACDE and EBCDA. After being primed with 10 samples of the ABCDE sequence, the model is able to **reject long subpaths** (CED, BAC, EB, DA) while **consolidating** the **short subpaths** (ABC, BCD, CDE) into the ABCDE sequence.

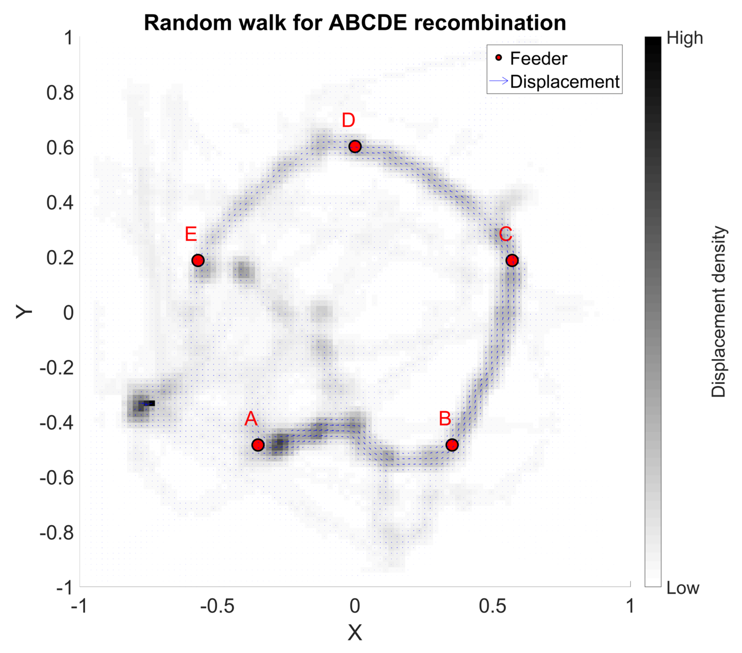

Spatial overlap is required

For a given feeder configuration, 9 different navigation trajectories contain subpaths of the target sequence. **Subpaths** are constrained to span over **2 feeders** in one condition and **3 feeders** in the other condition. Between each of the 9 first trials, the model is trained with snippets of the corresponding sequence. At trial 10, the model is exposed to snippets of the target sequence. The model is able to **discover** the target sequence before trial 10 only when snippets span subpaths of 3 feeders.

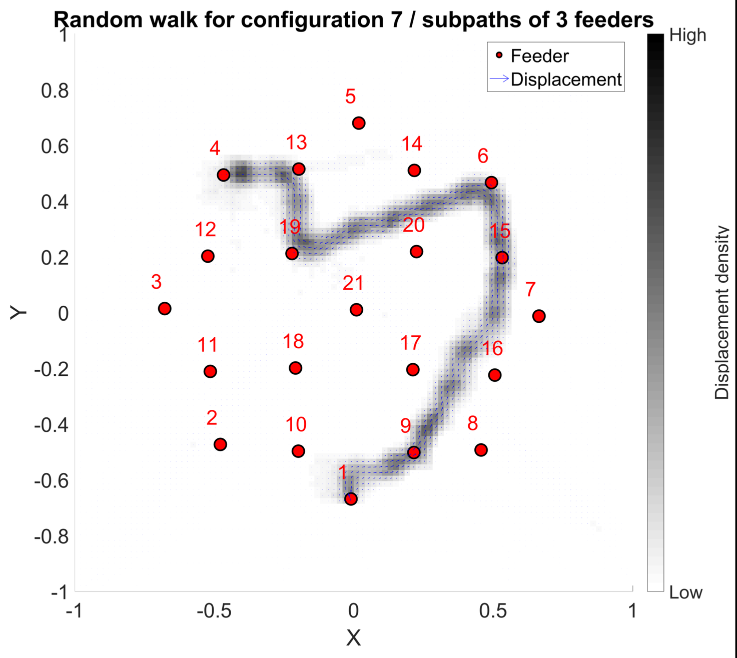

Condition	2 feeders subpaths	3 feeders subpaths
Config 6	Trial 10	Trial 4
Config 7	Trial 10	Trial 7

Conclusion

We demonstrated that the **TRN** reservoir model is able to **consolidate** randomly replayed snippets into efficient **navigation sequence**. It naturally **aligns** overlapping subsequences of stimuli in the reservoir **state space**. The limited **time budget** allocated to the random replay of snippets gives to the model the ability to **select** shortest paths between **reward** sites. Based on these two dynamical properties, we showed that the model is able to **consolidate** snippets of partially overlapping **multiple navigation sequences** in order to autonomously **generate novel efficient navigation sequences**. The current navigation model exploits a **spatial filter** to reduce noise in place cell predictions. It is based on place cells spatial response and it simulates a **perception/action** paradigm. The next version of the model will be implemented in a simulated and physical robotic **animat** that will take into account **embodiment** constraints. The online snippet replay model will be based on **electrophysiological** measurement of a rat **hippocampus**.

References

[1] Alejandra Barrera, Gonzalo Tejera, Martin Llofriu, and Alfredo Weitzenfeld. Learning spatial localization: from rat studies to computational models of the hippocampus. Spatial Cognition & Computation, 15(1):27-59, 2015.

[2] Laurel Watkins de Jong, Brian Gereke, Gerard M Martin, and Jean-Marc Fellous. The traveling salesrat: insights into the dynamics of efficient spatial navigation in the rodent. Journal of Neural Engineering, 8(6):065010, 2011.

[3] Peter Ford Dominey. Complex sensory-motor sequence learning based on recurrent state representation and reinforcement learning. Biological cybernetics, (73):265-274, 1995.

[4] Zhang, K., Ginzburg, I., McNaughton, B.L. & Sejnowski, T.J. Interpreting neuronal population activity by reconstruction: unified framework with application to hippocampal place cells. *Journal of neurophysiology* 79, 1017-44 (1998).

Acknowledgements

Supported by NSF-ANR CRCNS project **Spaquence** ANR-14-NEUC-0005-1
